# PRKCI Mediates Radiosensitivity *via* the Hedgehog/GLI1 Pathway in Cervical Cancer

**DOI:** 10.3389/fonc.2022.887139

**Published:** 2022-06-16

**Authors:** Zhuna Wu, Chunxian Huang, Ruixin Li, Hui Li, Huaiwu Lu, Zhongqiu Lin

**Affiliations:** ^1^ Department of Gynecological Oncology, Sun Yat-Sen Memorial Hospital, Sun Yat-Sen University, Guangzhou, China; ^2^ Guangdong Provincial Key Laboratory of Malignant Tumor Epigenetics and Gene Regulation, Sun Yat-sen Memorial Hospital, Sun Yat-sen University, Guangzhou, China; ^3^ Department of Gynecology and Obstetrics, The Second Affiliated Hospital of Fujian Medical University, Fujian Medical University, Quanzhou, China

**Keywords:** PRKCI, radiosensitivity, Hedgehog/GLI1 pathway, cervical cancer, auranofin

## Abstract

**Objective:**

Insensitivity to radiotherapy accounts for the majority of therapeutic failures in cervical cancer (CC) patients who undergo radical radiotherapy. We aimed to elucidate the molecular mechanisms underlying radiosensitivity to identify methods to improve the overall 5-year survival rate. The atypical protein kinase C iota (aPKCι) gene PRKCI exhibits tumor-specific copy number amplification (CNA) in CC. We investigated how PRKCI decreases radiosensitivity in CC and assessed the interplay between PRKCI and the Hedgehog (Hh)/GLI1 pathway in the present research.

**Methods:**

The biological functions of PRKCI in CC radiosensitivity were explored through immunohistochemistry, colony formation, Cell Counting Kit-8 (CCK-8), cell cycle, apoptosis assays, and xenograft models. qRT-PCR, Western blotting analysis, and immunofluorescence assays were utilized to evaluate the interplay between PRKCI and the Hh/GLI1 pathway and its mechanism in PRKCI-decreased radiosensitivity in CC. Furthermore, the effect of auranofin (AF), a selective inhibitor of PKCι, on CC cells was explored through biochemical assays *in vitro* and *in vivo*.

**Results:**

We found that high PRKCI expression was responsible for decreased survival in CC. PRKCI was intimately associated with radiation-triggered alterations in proliferation, the cell cycle, apoptosis, and xenograft growth. The Hh/GLI1 pathway was activated when PRKCI expression was altered. PRKCI functions downstream of the Hh/GLI1 pathway to phosphorylate and activate the transcription factor GLI1. AF acts as a radiosensitizer and showed biological effects *in vitro* and *in vivo*.

**Conclusions:**

PRKCI is a therapeutic target for regulating radiosensitivity in CC. This molecule regulates radiosensitivity by modulating GLI1 relocalization and phosphorylation in CC *via* the Hh/GLI1 pathway.

## Introduction

Cervical cancer (CC) is one of the most common malignant tumors in the female reproductive tract, with an age of onset of 50–55 years; approximately 1/4 of the new cases each year occur in China. Surgery and radiation are the primary treatments for CC, and approximately 80% of CC patients, especially advanced-stage patients, require radiation, which is the main treatment strategy. Although early-stage patients have a good prognosis, the 5-year survival rate is less than 20% after radiotherapy in advanced-stage patients, and decreased radiosensitivity is one of the primary reasons for treatment failure in CC patients (1). Radiosensitivity has a critical role in subjects who undergo radical radiotherapy (2). Although radiosensitivity is being intensively investigated, there is no effective method to enhance radiosensitivity in CC. Thus, exploration of the molecular mechanism of radiosensitivity and improvement of the 5-year survival rate of CC are urgently needed.

Copy number variants (CNVs) are common genetic variants in cancers, and the CNV burden is related to recurrence and death in various tumors (3). Copy number loss (CNL) and copy number amplification (CNA) are two types of CNVs. CNA of chromosome 3q26 was found to be the most common CNV in various tumors and was significantly associated with malignant transformation, metastasis, and poor clinical prognosis of CC, lung squamous cell carcinoma (LSCC), esophageal squamous cell carcinoma (ESCC), and head and neck squamous cell carcinoma (HNSCC) (4–9). Previous studies reported that 3q26 CNA is a common chromosomal abnormality that results in the progression of normal cervical epithelium to dysplasia (9) and dysplasia to invasive cancer (6) and exists in 77%–90% of CC cases. The chromosome 3q26 region contains approximately 200 protein-encoding genes.

PRKCI is part of the 3q26 amplicon that overexpresses protein kinase C iota (PKCι) and frequently becomes oncogenic through CNA in some tumors 10; 11, 12). PRKCI encodes PKCι, which is an atypical subclass in the PKC gene family. PKCι is overexpressed and associated with poor prognosis in many tumors, especially in advanced malignant tumors (13–15). Overexpression of PKCι promotes the transformation of the normal cervical epithelium into invasive cancer (16, 17). PKCι influences the polarity and fate of epithelial cells and tissue integrity of untransformed cells *via* subcellular localization (18).

In LSCC, PKCι expression is driven by PRKCI CNG/CNA, and a novel PKCι-dependent Hedgehog (Hh) pathway was shown to influence the transformation and growth of cells (19). Inhibition of the Hh pathway increased the radiosensitivity of basal cell carcinoma (BCC) and HNSCC cells (20). In mouse xenograft models of CC and esophageal adenocarcinoma, Hh inhibition enhances radiosensitivity (21, 22). However, to what extent PRKCI is related to CC radiosensitivity and how PRKCI influences the mechanisms of radiosensitivity remain unclear, and we aimed to confirm the radiosensitizing effect of PRKCI in CC.

## Materials and Methods

### The Cancer Genome Atlas Bioinformatics Analyses

Transcriptome RNA-seq data and the CNA status of PRKCI in The Cancer Genome Atlas (TCGA) cervical squamous cell carcinoma and endocervical adenocarcinoma (CESC) samples were analyzed with cBioPortal software. A total of 319 tissue samples (normal and tumor), 309 paired samples (normal and CESC), and matching clinicopathologic data were downloaded from TCGA CESC dataset. Gene count values were ranked according to PRKCI expression and compared to assess the differential expression of HHAT, SMO, and GLI1.

### Patient and Tissue Samples

A total of sixty paraffin-embedded CC specimens were obtained at The Second Affiliated Hospital of Fujian Medical University (Fujian, China) from October 2015 to October 2019. The main treatment for all patients was radical radiotherapy. The date cutoff was July 2021, the follow-up time was 2–63 months (median 27 months), and details about cancer-related death and recurrence were collected. The research was approved by the Research Ethics Committee of The Second Affiliated Hospital of Fujian Medical University prior to the study. The research was conducted based on the Declaration of Helsinki.

### Immunohistochemistry

Immunohistochemistry (IHC) staining was performed as previously described (23). The primary antibodies included anti-PKCι (Abcam, Inc., Cambridge, UK), anti-Smoothened (Abcam, Inc., Cambridge, UK), anti-GLI1 (Abcam, Inc., Cambridge, UK), anti-HHAT (Novus Biologicals, Littleton, CO, USA), and anti-Ki-67 (ProteinTech, Chicago, IL, USA). The secondary antibodies included anti-rabbit IgG (Cell Signaling Technology, Danvers, MA, USA). The proportion of PRKCI, SMO, and GLI1 staining was scored as follows: less than 1/3 = 1, between 1/3 and 2/3 = 2, or more than 2/3 = 3. The staining intensity was scored as follows: negative = 0, light yellow = 1, brownish yellow = 2, or tan = 3. The final score for PRKCI, SMO, and GLI1 expression was calculated by multiplying the 2 scores. The slides were classified into low- and high-expression groups, corresponding to scores of <6 and ≥6, respectively. The histopathological diagnosis of the patients included in our study was established by two pathologists who specialized in gynecologic oncology.

### Cell Lines and Cell Culture

Two CC cell lines (HeLa and SiHa) were purchased from GeneChem (Shanghai, China). The cell culture medium was Dulbecco’s modified Eagle’s medium (DMEM) (Gibco, Grand Island, NY, USA) with 10% fetal bovine serum (FBS; Biological Industries, Biological Industries, Israel South America), and the cells were cultured at 37°C with 5% CO_2_ in humidified air.

### Transfection With ShRNA

PRKCI shRNA and negative control shRNA were incorporated into the lentiviral vector GV248, and the full-length PRKCI sequence was incorporated into the lentiviral vector GV358, with the empty vector as a control. Following the manufacturer’s protocol, HeLa and SiHa cells were stably transfected with PRKCI shRNA (GeneChem, Shanghai, China) and selected by puromycin. All stable cell lines were created with transfection of infection with lentiviruses. The sequences of shRNA are recorded in **Table S1**. The protocol of cell transfection was described previously (24). qRT-PCR and Western blotting assays were used to assess the mRNA and protein expression of PRKCI when downregulated and upregulated by lentiviral vectors in both HeLa and SiHa cells (**Figures S1A, B, C**).

### Small Interfering RNA Transfection

Cells were transfected with small interfering RNA (siRNA)-con or with siRNA-GLI1. The sequences of siRNA-GLI1 (GenePharma, Shanghai, China) is listed in **Table S2**. According to the manufacturer’s protocol, transfections were performed with Lipofectamine 3000 reagent (Thermo Fisher, Waltham, MA, USA). qRT-PCR and Western blotting assays were applied to assess the reduction in GLI1 mRNA and protein expression 48 and 72 h after transfection (**Figures S1D, E**). Cell Counting Kit-8 (CCK-8) assays were performed 24 h after transfection.

### Colony Formation Assays

The cells were counted and plated in 6-well plates at different densities (1 × 10^3^, 2 × 10^3^, 4 × 10^3^, 8 × 10^3^, or 1.2 × 10^4^ cells per well) and irradiated with various doses (0, 2, 4, 6, and 8 Gy) after 24 h. The cells were stained and fixed after 14 days when colonies were visible. Viable colonies, reported as those with 50 or more cells, were counted. The plating efficiency (PE) was calculated as the number of colonies/the number of inoculated cells × 100%. The survival fraction (SF) was calculated as the PE of the test group divided by the PE of the 0 Gy group.

### Cell Counting Kit-8 Assay

A final concentration of 100 µl/ml of CCK-8 reagent (APExBIO, Houston, TX, USA) was added, and the cells were incubated at 37°C for 2 h. Absorbance values at 450 nm were detected. CCK-8 assays were performed 24 h after 8-Gy irradiation.

### Cell Apoptosis

The cells were resuspended in 1× annexin V binding working solution and then stained with annexin V-APC and 7-ADD (Elabscience, Wuhan, China) for 30 min at room temperature. A flow cytometer (BD FACSCalibur, BD Biosciences, San Jose, CA, USA) was used. The apoptosis assay was performed 48 h after 8-Gy irradiation.

### Cell Cycle Analysis

The cells were fixed in 70% cold ethyl alcohol at 4°C overnight. Then, the cells were washed and suspended in 500 µl of staining buffer containing 10 µl of RNase (100 µg/ml) and 25 µl of propidium iodide (PI) (Beyotime, Nanjing, China) and incubated for 30 min in the dark. Subsequently, Cell Quest software (BD Biosciences, USA) was used to test the cell cycle phases. Cell cycle analysis was performed 48 h after 8-Gy irradiation.

### Immunofluorescence Staining Assay

Cells were plated in confocal dishes and subjected to 8-Gy irradiation. After 48 h, the cells were fixed in 4% paraformaldehyde for 20 min, permeabilized with 0.5% Triton for 20 min, blocked with 1% bovine serum albumin (BSA) for 1 h, and incubated with anti-GLI1 antibody (1:300, Abcam, Cambridge, MA, USA) at 4°C overnight and Cy3-labeled secondary antibody (1:100, goat anti-rabbit IgG, Boster, Wuhan, China). The dishes were scanned with a laser confocal scanning microscope (Zeiss LSM710, Carl Zeiss, Oberkochen, Germany).

### Quantitative Real-Time PCR

Total RNA was extracted from cell lines utilizing an RNA-Quick purification kit (Yishan Biotechnology Co., Shanghai, China), and then, cDNA was prepared according to the manufacturer’s protocol (TaKaRa, Maebashi, Japan). qRT-PCR was performed with a CFX ConnectTM instrument (Bio-Rad, Hercules, CA, USA). The detailed procedure is presented in the Supplementary Methods. The relative mRNA expression of PRKCI, HHAT, SMO, and GLI1 was calculated by the 2^−ΔΔCt^ method. The primer sequences are shown in **Table S3**. qRT-PCR for each sample was repeated in three independent experiments.

### Western Blotting

Total protein was extracted from cells using radioimmunoprecipitation assay (RIPA) lysis buffer (CW Biotechnology, Beijing, China) with protease and phosphatase inhibitors (Solarbio, Beijing, China; and CW Biotechnology, Beijing, China). According to the manufacturer’s protocol, nuclear and cytosolic lysates were extracted by using the Nuclear and Cytoplasmic Protein Extraction Kit (Beyotime, China). Western blotting was performed as described previously (25). The following antibodies were utilized: anti-PKCι (Abcam, Inc., Cambridge, UK), anti-Smoothened (Abcam, Inc., Cambridge, UK), anti-GLI1 (Abcam, Inc., Cambridge, UK), anti-HHAT (Novus Biologicals, USA), anti-pGLI1 (Thr1074) (Affinity Biosciences, Cincinnati, OH, USA), and anti-GAPDH (Cell Signaling Technology, USA). The secondary antibodies included anti-rabbit IgG (Cell Signaling Technology, USA).

### Xenograft Experiment

Female BALB/c nude mice (4–5 weeks old) were used for the CC xenograft model and were obtained from the Laboratory Animal Center (Sun Yat-Sen University, China). First, a suspension of 5 × 10^6^ SiHa cells was inoculated subcutaneously into the right axillary region (n = 5 per group). Subsequently, 1 week after inoculation, the mice were subjected to a locoregionally applied body dose of 6 Gy every week using an X-RAD irradiator (Rad Source 2000, Rad Source, Buford, GA, USA). Tumor size was measured every 4 days, and tumor volume was calculated with the following formula: volume = width^2^ × length × 0.5. After the 28-day experiment, all mice were sacrificed, and the tumors were excised, harvested, and weighed. A portion of each tumor was embedded for IHC studies. All animal experimental procedures and care were approved by the Animal Experiment Ethics Committee (Sun Yat-Sen University, China).

### Auranofin Experiments

Auranofin (AF) was purchased from APExBIO. Solid AF was diluted in dimethyl sulfoxide (DMSO) at the indicated concentrations (final concentration of 0.1% DMSO). HeLa and SiHa cells were treated with 0, 0.25, 0.5, 1, 2, or 4 µM AF for 48 h. The half-maximal inhibitory concentration (IC50) values of AF were determined by CCK-8 assays. The IC50 values were calculated with GraphPad 8.0 software. The IC50 values of AF were used for subsequent drug experiments, including colony formation, CCK-8, cell apoptosis, and cell cycle assays. HeLa and SiHa cells were treated with AF 2 h before 8-Gy irradiation. The detailed procedure of the colony formation and xenograft experiments is presented in the **Supplementary Methods**.

### Statistical Analysis

All statistical analyses were performed with GraphPad Prism 8.0, except for univariate and multivariate Cox regression analyses, which were performed with SPSS Statistics version 26.0 (IBM, Armonk, NY, USA). All statistical methods are shown in the figure legends. Data are presented as the mean or mean ± SD. Differences with p < 0.05 were considered statistically significant. All experiments were independently repeated in triplicate.

## 3. Results

### 3.1 Genetic Alterations Result in the Upregulation of PRKCI mRNA Expression

**Figure 1:**
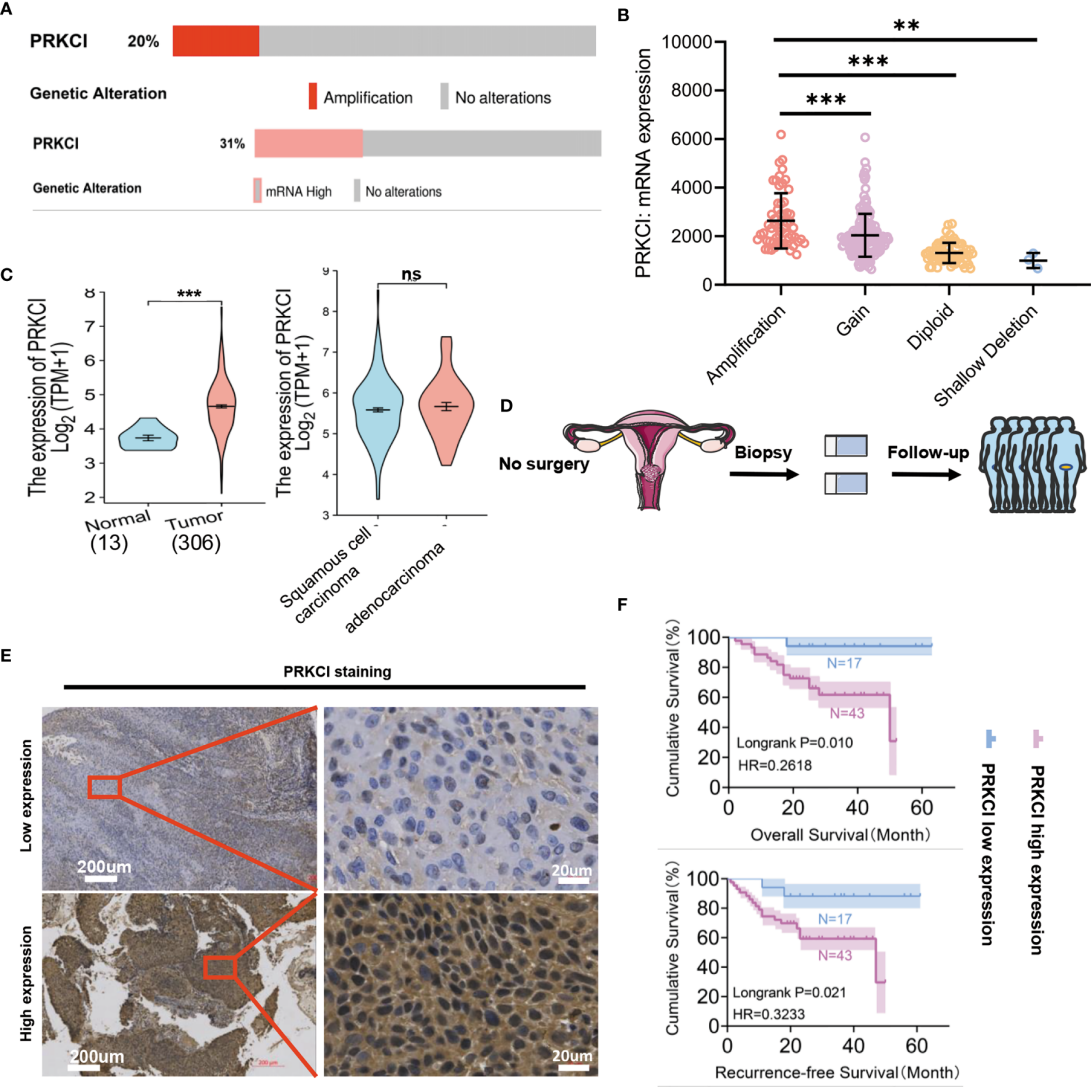
Identification of PRKCI alterations in CC. **(A)** PRKCI amplification (top) and mRNA overexpression (bottom) in CC (TCGA dataset). Red bars, tumors with amplification (top); pink bar, tumors with overexpression (bottom); gray bars, tumors with no alterations in CNA (top) or expression (bottom). **(B)** Differences in PRKCI mRNA expression between the amplified and non-amplified groups in TCGA CESC samples. **(C)** Significantly elevated PRKCI expression was observed in CC tissues compared with normal specimens (TCGA dataset) (n = 319). No differences in PRKCI expression between the two pathotypes were observed. **(D)** Workflow for PRKCI identification in patients who underwent radical radiotherapy. **(E)** Representative images (×50 and ×400) of IHC staining for PRKCI in 60 CC patients who underwent radical radiotherapy (high expression vs. low expression). **(F)** Radical radiotherapy-treated CC patients with high PRKCI expression had shorter recurrence-free survival (RFS) and overall survival (OS) than patients with low PRKCI expression (n = 60). Scale bars are shown. Data are shown as the mean ± SD (**B**, **C**). ***p < 0.001, **p < 0.01; ns, not significant. p-Values were calculated by the log-rank test **(F)**. CC, cervical cancer; TCGA, The Cancer Genome Atlas; CNA, copy number amplification; CESC, endocervical adenocarcinoma; IHC, immunohistochemistry.

The genetic CNA and mRNA expression profiles of PRKCI were obtained from TCGA *via* cBioPortal for Cancer Genomics (cBioPortal) (http://www.cbioportal.org/). Gene CNA of PRKCI was found in 20% (altered/profiled = 60/295) of the sequenced samples. PRKCI mRNA expression was found in 31% (altered/profiled = 93/304) of the patients (**Figure 1A**). The mRNA expression of the PRKCI gene was higher in the amplified group in TCGA CESC samples (mean ± SD: amplification vs. gain, 2,637 ± 1,137 vs. 2,036 ± 884.7, p < 0.001; amplification vs. diploid, 2,637 ± 1,137 vs. 13,126 ± 415.4.7, p < 0.001; amplification vs. shallow deletion, 2,637 ± 1,137 vs. 999.1 ± 312.9, p < 0.01) (**Figure 1B**). The PRKCI mRNA level was significantly higher in the tumor group (N = 306) than in the normal group (N = 13) (4.66 ± 0.769 vs. 3.736 ± 0.295, p < 0.001), but no significant differences were found between the squamous cell carcinoma and adenocarcinoma CC samples (**Figure 1C**). Based on the above results, we hypothesized that genetic alterations resulted in the upregulation of PRKCI mRNA expression in CC.

### 3.2 PRKCI Is Significantly Highly Expressed in Cervical Cancer Tissue and Is Associated With Pathological Grade and Prognosis of Cervical Cancer Patients

A schematic of the clinical study flowchart is shown (**Figure 1D**). To determine the clinical importance of PRKCI in CC, we detected PRKCI protein levels in 60 samples from CC patients who underwent radiotherapy as a primary treatment regimen. Among them, 43 samples (71.67%) had high PRKCI expression, and 17 (28.33%) had low PRKCI expression (**Figure 1E**). The data showed significantly high expression of PRKCI in patients with an advanced tumor grade, recurrence, and CC-related death (**Table 1**). Moreover, survival analysis revealed that high PRKCI expression was strongly associated with poor survival (log-rank test, p = 0.010; **Figure 1F**) and shorter recurrence-free time (log-rank test, p = 0.021; **Figure 1F**). The median survival and median recurrence interval times of the high-expression group were 50.0 and 47.0 months, respectively, and the survival curve was not lower than 50% in the low-expression group. Furthermore, univariate analysis revealed that high PRKCI expression, tumor grade, and age were significantly related to an increased risk of cancer-specific death (**Table 2**). The multivariate analysis showed that high PRKCI expression and tumor grade were significant prognostic factors (**Table 2**). In conclusion, high PRKCI expression was significantly related to poor overall survival in CC patients who underwent radiotherapy independent of tumor grade and age (p = 0.032, **Table 2**). The above data confirmed our earlier hypothesis that PRKCI is significantly overexpressed in CC and is an independent factor for predicting the prognosis of CC patients who are insensitive to radiotherapy.

**Table 1:** Associations between PRKCI expression and clinicopathologic characteristics in CC.

Variable	Case			Low PRKCI expression			High PRKCI expression			p-Value^†^
	N	(%)		N	(%)		N	(%)		
Age (years)^‡^										
≤55	31	51.67		10	58.82		21	48.84		0.485
>55	29	48.33		7	41.18		22	51.16		
FIGO stage										
I–II	38	63.33		11	64.71		27	62.79		0.889
III–IV	22	36.67		6	35.29		16	37.21		
Histologic subtype										
Squamous	57	95.00		15	88.24		42	97.67		0.130
Adenocarcinoma	3	5.00		2	11.76		1	2.33		
Tumor grade										
G1/G2	50	83.33		17	100.0		33	76.74		0.029^*^
G3	10	16.67		0	0.00		10	23.26		
Recurrence										
No	40	66.67		15	88.24		25	58.14		0.026^*^
Yes	20	33.33		2	11.76		18	41.86		
Cervical cancer-related death										
No	43	71.67		16	94.12		27	62.79		0.015^*^
Yes	17	28.33		1	5.88		16	37.21		

N, number; FIGO, International Federation of Gynecology and Obstetrics; CC, cervical cancer.

^†^Chi-square test, *p < 0.05.

^‡^Range 32–76 years, median 55 years.

**Table 2:** Univariate and multivariate Cox regression analyses of overall survival in CC patients who underwent radiotherapy (n = 60).

Variable	Univariate analysis				Multivariate analysis		
	HR	95% CI	p-Value		HR	95% CI	p-Value
Age	1.052	1.003–1.104	0.038^*^		1.051	1.000–1.105	0.051
FIGO stage (I, II vs. III, IV)	0.909	0.329–2.510	0.854		1.181	0.424–3.287	0.750
Histologic subtype	0.463	0.000–187.386	0.463		0.000	0.000–	0.988
Tumor grade (G1, G2 vs. G3)	0.102	0.013–0.768	0.027^*^		0.108	0.014–0.828	0.032^*^
PRKCI expression	0.107	0.014–0.825	0.032^*^		0.106	0.014–0.831	0.033^*^

HR, hazard ratio; CC, cervical cancer; FIGO, International Federation of Gynecology and Obstetrics.

Cox regression analysis, *p < 0.05.

### 3.3 Regulation of PRKCI Expression Changed the Sensitivity of Cervical Cancer to Irradiation *In Vitro*

**Figure 2:**
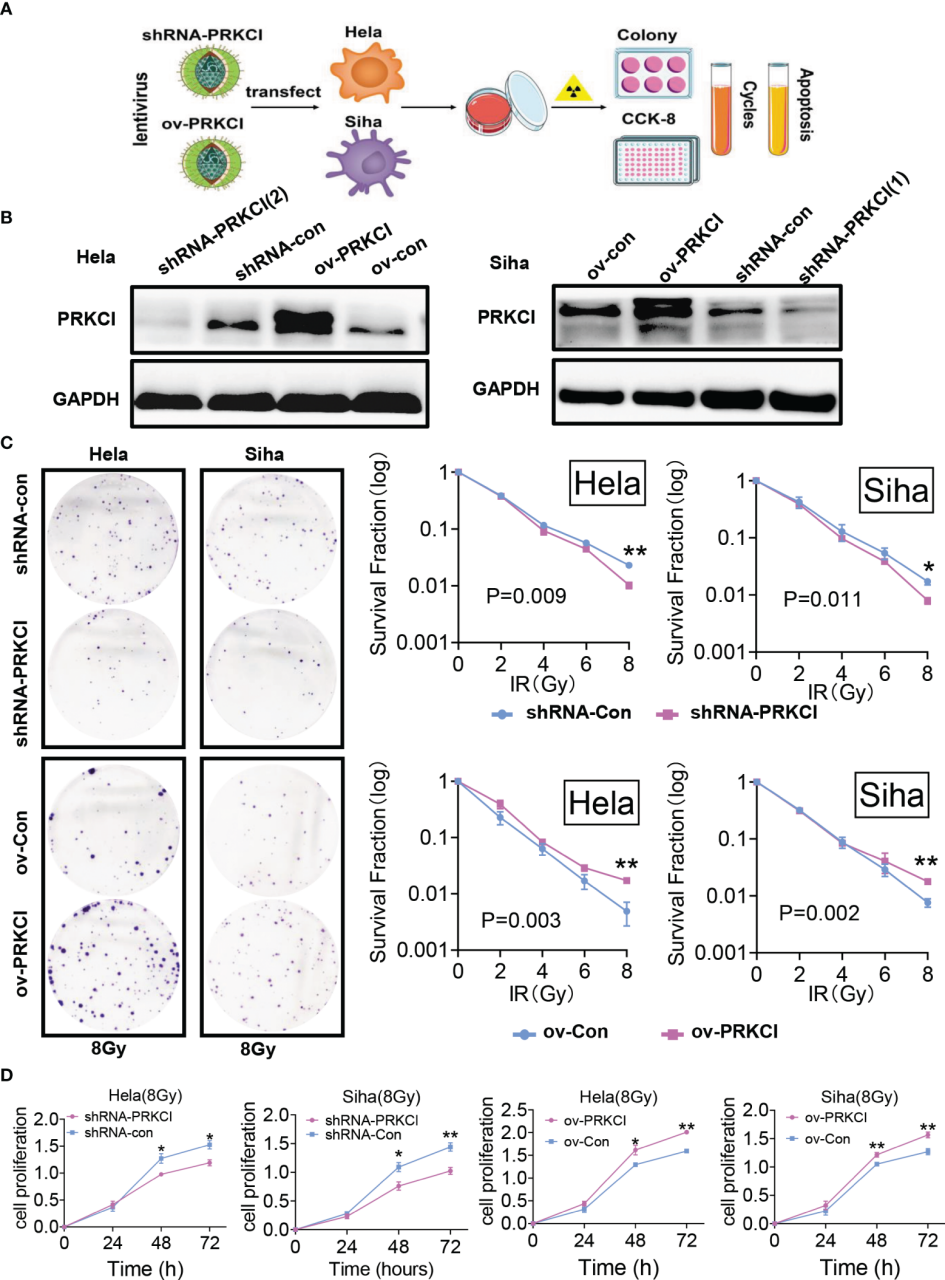
Influence of PRKCI suppression and overexpression on radiosensitivity in CC cells in cell proliferation. **(A)** Workflow for PRKCI regulation and research of CC cells. **(B)** The shRNA-PRKCI and ov-PRKCI cells were verified for the downregulation and upregulation of HeLa and SiHa cells by Western blotting analysis. **(C)** In colony formation assays, the proliferation of the shRNA-PRKCI- and ov-PRKCI-transfected HeLa and SiHa cells was affected by radiotherapy in a dose-dependent manner. **(D)** The viability of the shRNA-PRKCI- and ov-PRKCI-transfected HeLa and SiHa cells after radiotherapy at 8 Gy were determined by CCK-8 assays. Data are shown as the mean ± SD **(C, D)**. *p < 0.05; **p < 0.01 by multiple t-tests **(C, D)**. CC, cervical cancer; CCK-8, Cell Counting Kit-8.

**Figure 3:**
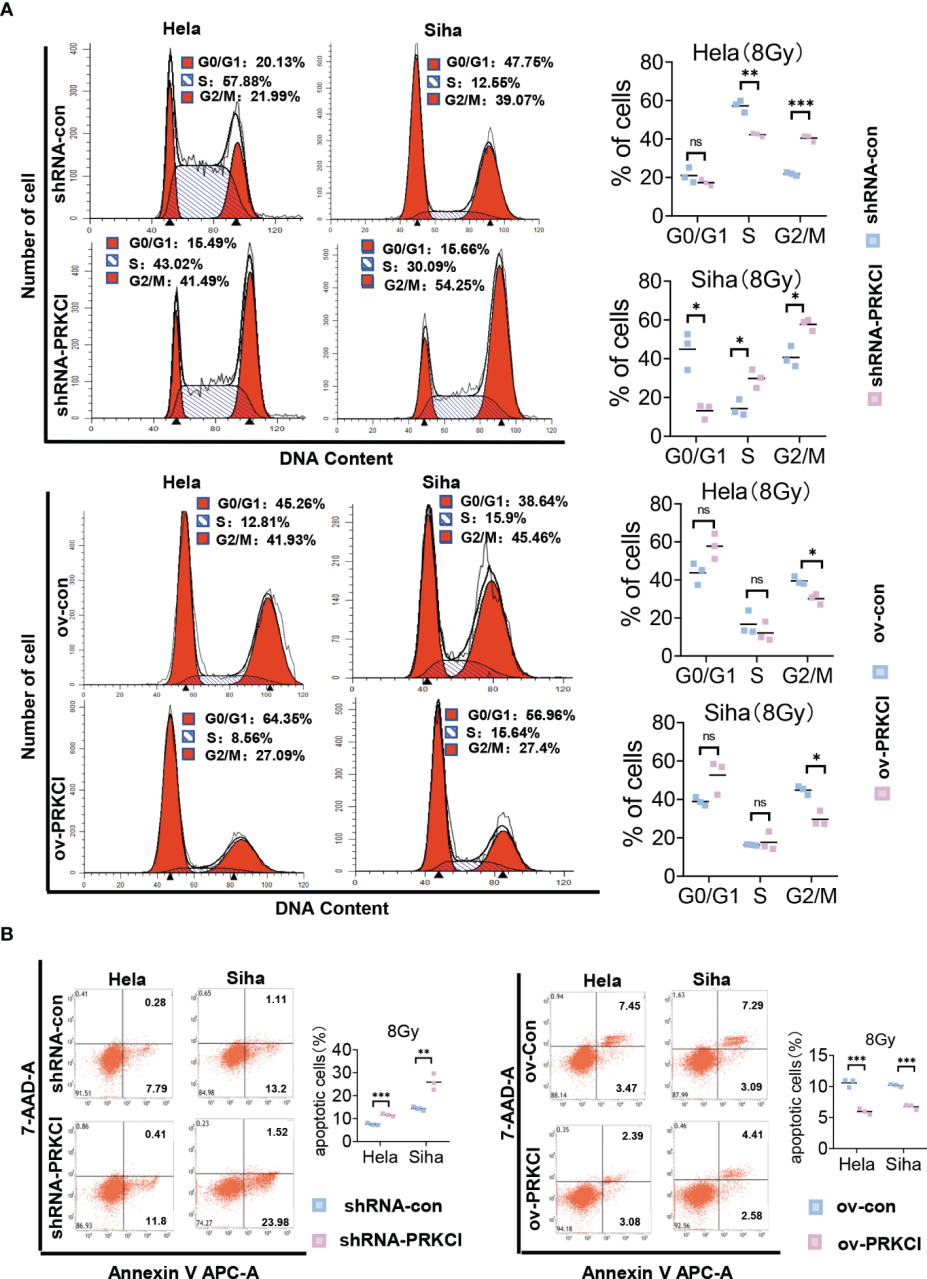
Influence of PRKCI suppression and overexpression on radiosensitivity in CC cells in cell cycle and apoptosis. **(A)** PRKCI decreases the G2/M population of CC cells treated with radiotherapy. **(B)** PRKCI decreases apoptosis of CC cells treated with radiotherapy. Data are shown as the mean from three independent experiments **(A, B)**. *p < 0.05; **p < 0.01; ***p < 0.001 by unpaired t-tests **(A, B)**. ns, not significant. CC, cervical cancer.

We investigated the role of PRKCI in CC cell radiosensitivity *in vitro*. A flowchart of the *in vitro* study process is shown (**Figure 2A**). The shRNA-PRKCI and ov-PRKCI cells were screened and verified for downregulation and upregulation of PRKCI expression in HeLa and SiHa cells by Western blotting (**Figure 2B**). The colony formation assay revealed that downregulation of PRKCI expression significantly decreased the number of colonies formed by CC cells, while overexpression of PRKCI substantially increased the number of colonies (**Figure 2C**). Downregulation of PRKCI expression resulted in a notable decrease in HeLa and SiHa cell viability after exposure to 8-Gy irradiation, as determined by CCK-8 assays (shRNA-con cells as the control), whereas upregulation of PRKCI expression had the opposite effect (ov-con cells as the control) (**Figure 2D**). Furthermore, the downregulation of PRKCI expression increased G2/M phase arrest, whereas the upregulation of PRKCI expression decreased G2/M phase arrest 48 h after 8-Gy irradiation (**Figure 3A**). Similarly, markedly increased apoptosis rates were observed in the HeLa-shRNA-PRKCI and SiHa-shRNA-PRKCI cells compared with the control cells, whereas upregulation of PRKCI expression had the opposite effect 48 h after 8-Gy irradiation (**Figure 3B**). However, the CCK-8 assay, apoptosis assay, and cell cycle assay showed that the downregulation and upregulation of PRKCI expression in HeLa and SiHa cells had no effect without irradiation (**Figures S2A, B**, **S3A, B**). These data and results showed that PRKCI is pivotal for the apoptosis, proliferation, and cell cycle of CC cells after irradiation but has no effect without irradiation. Therefore, these observations indicated that PRKCI regulates the sensitivity of CC cells to radiotherapy.

### 3.4 PRKCI Is Crucial for Xenograft Tumor Growth and Insensitivity to Irradiation *In Vivo*

**Figure 4:**
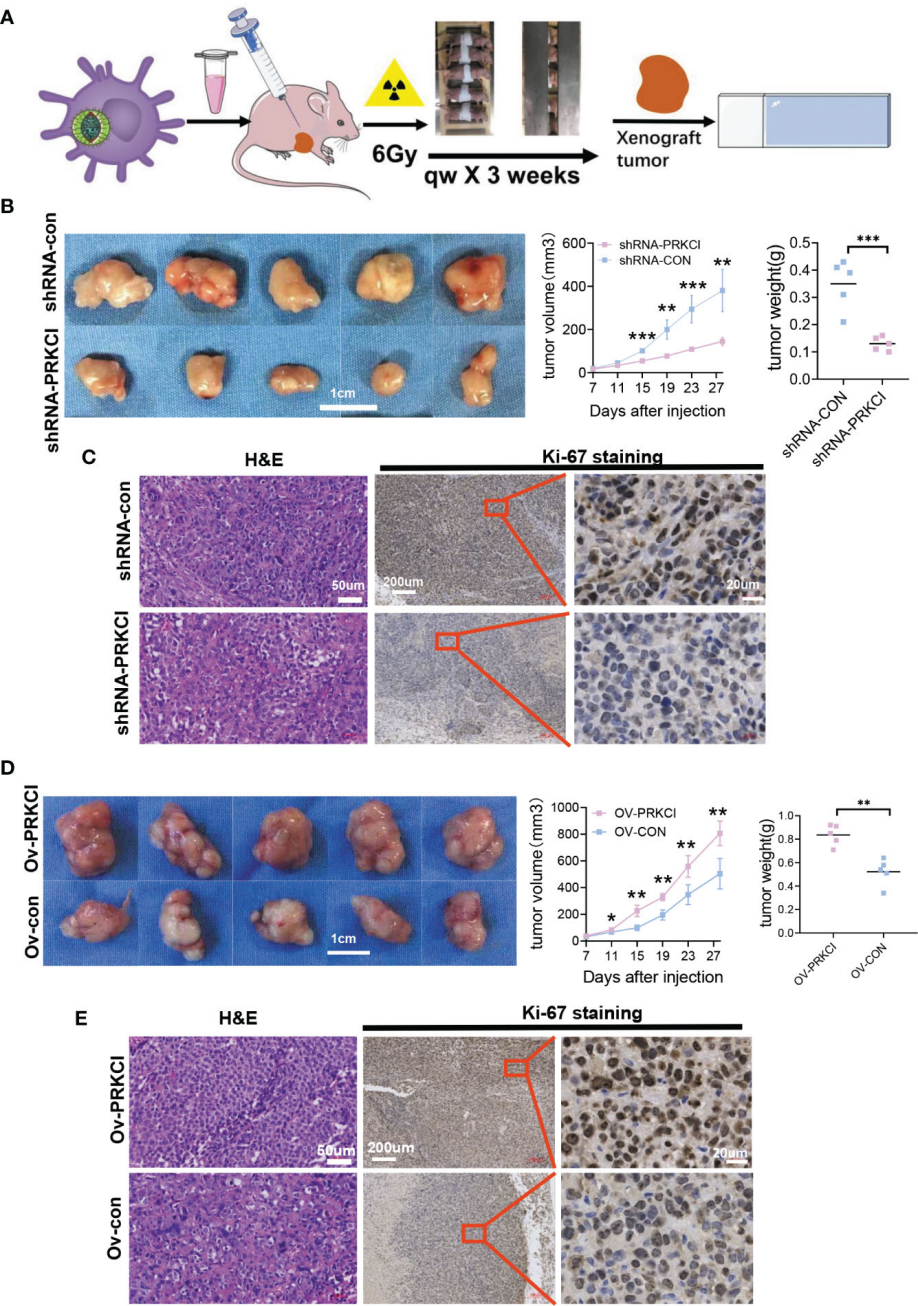
Influence of PRKCI suppression and overexpression on radiosensitivity in CC cells *in vivo*. **(A)** Workflow for nude mice with xenografts treated with 6 Gy/qw radiation for 3 weeks. **(B)** Nude mice with SiHa-shRNA-PRKCI and SiHa-shRNA-con were treated with radiation. The xenograft weights and volumes were measured (n = 5/group). **(C)** Representative images of H&E (×100) and Ki-67 (×50, ×400) staining of the xenografts. Ki-67 staining showed that PRKCI knockdown decreased proliferation. **(D)** Nude mouse models treated with SiHa-ov-PRKCI and SiHa-ov-con were presented and were treated with radiation. The xenograft weights and volumes were measured (n = 5/group). **(E)** Representative images of H&E (×100) and Ki-67 (×50, ×400) staining of the xenografts. Ki-67 staining showed that PRKCI overexpression increased proliferation. Scale bars are shown. Data are shown as the mean (n = 5). *p < 0.05; **p < 0.01; ***p < 0.001 by unpaired t-tests **(B, C, D)**, by multiple t-tests **(B, D)**. CC, cervical cancer.

Next, we further explored the role of PRKCI in xenograft tumor progression and sensitivity to radiotherapy. A flowchart of the *in vivo* study is shown (**Figure 4A**). BALB/c nude mice were subcutaneously injected with SiHa cells stably transfected with the ov-PRKCI or ov-con vectors. Consistent with the *in vitro* results, tumors in the shRNA-PRKCI group grew substantially slower and weighed less than those in the shRNA-con group after irradiation (**Figures 4B**, **S4A**). Furthermore, IHC staining demonstrated that tumors from the shRNA-PRKCI group exhibited significantly reduced Ki-67 staining as compared with those in the shRNA-con group (**Figure 4C**). Conversely, we found that the tumors in the ov-PRKCI group grew significantly quicker and were heavier than those in the ov-con group after irradiation (**Figures 4D**, S4A). IHC staining showed that tumors from the ov-PRKCI group exhibited significantly increased Ki-67 staining as compared with those from the ov-con group (**Figure 4E**). In summary, the above results revealed that PRKCI plays a pivotal role in regulating radiosensitivity *in vitro* and *in vivo* by altering irradiation-induced effects.

### 3.5 PRKCI Mediates Radiosensitivity *via* the Hh/GLI1 Pathway to Regulate GLI1 Relocalization and Phosphorylation in Cervical Cancer

**Figure 5:**
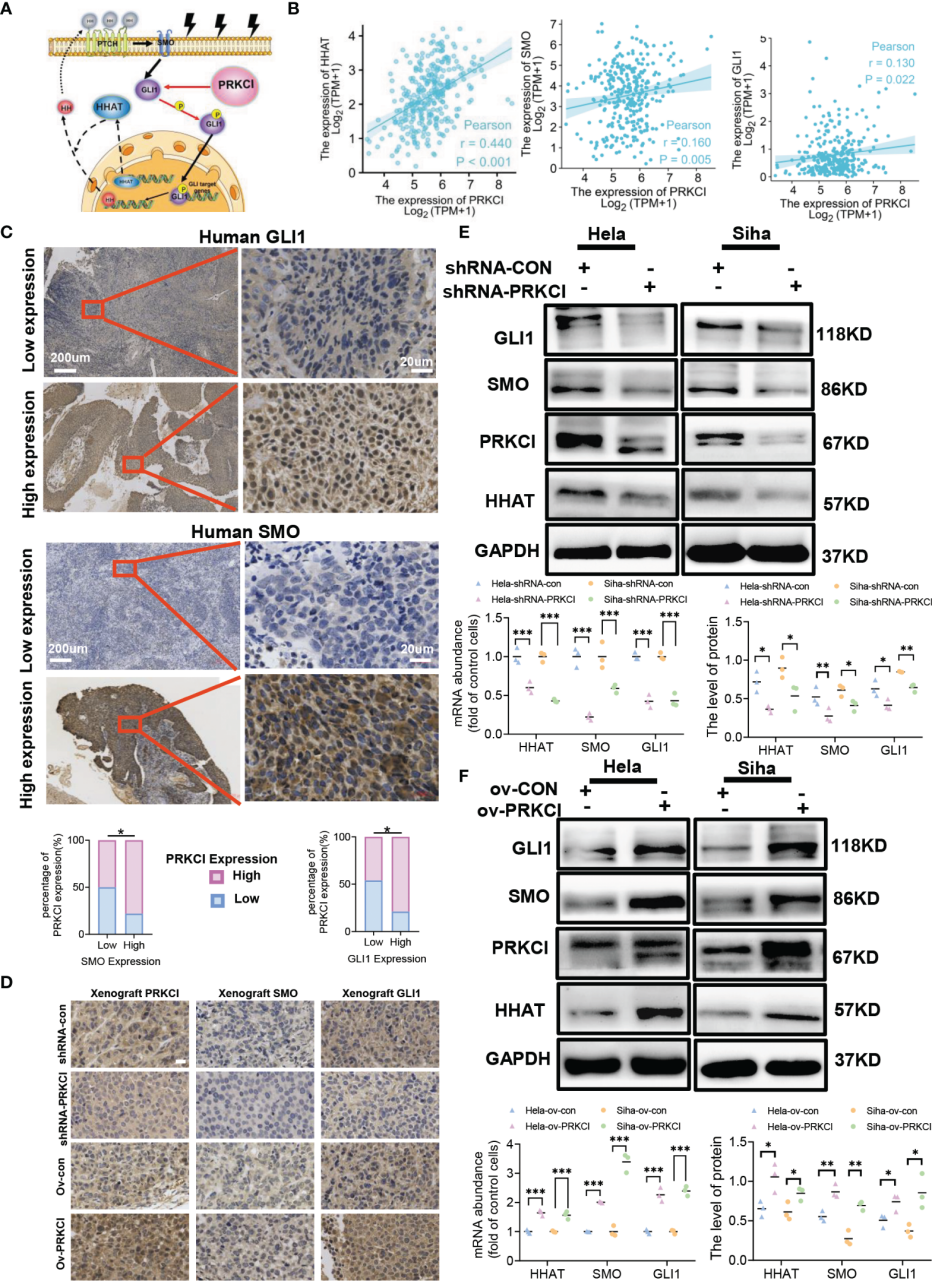
PRKCI mediated radiosensitivity in CC *via* the Hedgehog signaling pathway. **(A)** Schematic of the PRKCI and Hh signaling transduction pathways. **(B)** The association between PRKCI mRNA levels and HHAT, SMO, and GLI1 levels in CC samples (n = 306, TCGA dataset). **(C)** Representative images (×50 and ×400) of IHC staining for SMO and GLI1 in 60 CC patients who underwent radical radiotherapy (high expression vs. low expression). Correlation analysis for PRKCI, SMO, and GLI1 in patients (n = 60). **(D)** Representative images of PRKCI (×400), SMO (×400), and GLI1 (×400) staining of the xenografts from different groups; scale bar = 20 µm. **(E)** HeLa and SiHa cells were transfected with empty vector (GV248) or vector containing PRKCI shRNA and treated with radiation. The mRNA and protein levels of HHAT, SMO, and GLI1 are presented. **(F)** HeLa and SiHa cells were transfected with empty vector (GV358) or vector containing PRKCI and treated with radiation. The mRNA and protein levels of HHAT, SMO, and GLI1 are presented. Scale bars are shown. p-Values were calculated by Pearson’s test **(B)**. Data are shown as the mean ± SD from three independent experiments. *p < 0.05; **p < 0.01; ***p < 0.001 by chi-square tests **(C)**, by multiple t-tests **(E, F)**. CC, cervical cancer; TCGA, The Cancer Genome Atlas; IHC, immunohistochemistry.

aPKCι/λ is a potential target for the treatment of Hh-dependent and Smo inhibitor-resistant advanced BCC (26). SOX2 and PRKCI are coamplified and collaborate to trigger the Hh pathway in LSCC (19). aPKCι/λ mediates BCC growth by activating Gli (27). We further explored whether PRKCI is involved in the Hh/GLI1 pathway. A schematic diagram of the relationship between PRKCI and the Hh/GLI1 pathway is shown (**Figure 5A**). The link between PRKCI and the Hh/GLI1 pathway is functionally associated with these proteins (HHAT, SMO, and GLI1). We assessed whether these genes are consistently overexpressed in CC. Analysis of the CESC dataset (306 samples) from TCGA revealed modest positive correlations between PRKCI expression and HHAT, SMO, and GLI1, three key Hh pathway components mediated by PRKCI in CESC (**Figure 5B**).

**Figure 6:**
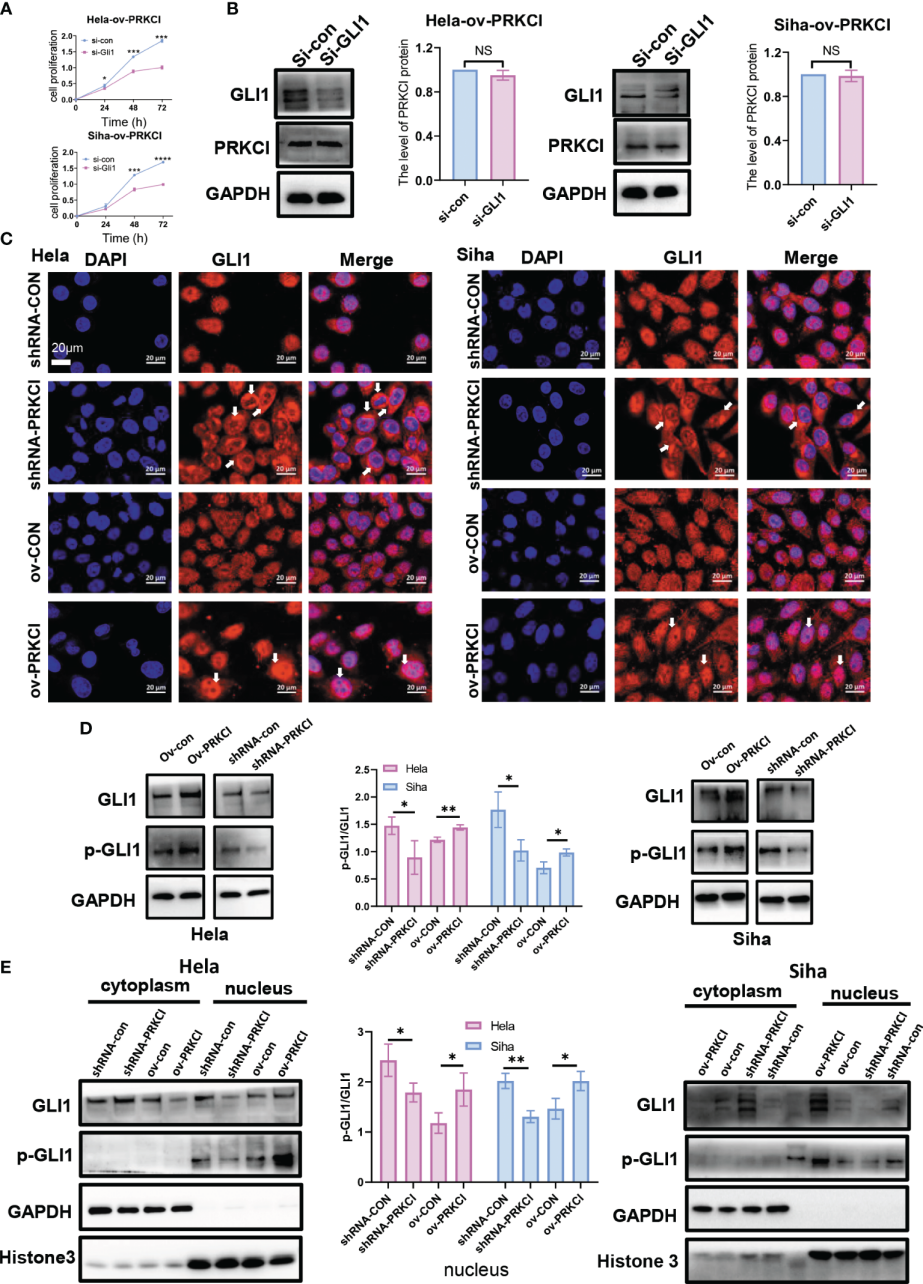
PRKCI mediated radiosensitivity in CC *via* the Hh signaling pathway to change GLI1 localization. **(A)** The viability of the si-GLI1-transfected HeLa-ov-PRKCI and SiHa-ov-PRKCI cells after radiotherapy at 8 Gy determined by CCK-8 assays. **(B)** Western blotting assays were used to assess the protein expression of PRKCI when GLI1 expression was downregulated by siRNA in both the HeLa-ov-PRKCI and SiHa-ov-PRKCI cells. **(C)** The relative protein localization of GLI1 after transfection with shRNA-con and shRNA-PRKCI or ov-con and ov-PRKCI in the HeLa and SiHa cells treated with radiation was determined by immunofluorescence assays. **(D)** Western blotting analysis of GLI1 and p-GLI1 protein levels in the shRNA-PRKCI- and ov-PRKCI-transfected HeLa and SiHa cells. **(E)** Western blotting analysis of GLI1 and p-GLI1 protein levels in the cytoplasmic and nuclear fractions of the shRNA-PRKCI- and ov-PRKCI-transfected HeLa and SiHa cells. Scale bars are shown. Data are shown as the mean ± SD from three independent experiments. NS, not significant. *p < 0.05; **p < 0.01; ***p < 0.001; ****p < 0.0001 by multiple t-tests **(A)**. CC, cervical cancer; CCK-8, Cell Counting Kit-8.

To validate the interplay between the Hh/GLI1 pathway and PRKCI, we performed IHC staining of SMO and GLI1. We detected the protein levels of SMO and GLI1 in 60 CC patients. In the previous cases, 46 samples (76.67%) had high SMO expression, 14 (23.33%) had low SMO expression, 47 (78.33%) had high GLI1 expression, and 13 (21.67%) had low GLI1 expression. These data demonstrated that PRKCI was correlated to SMO and GLI1 (rSMO = 0.434, p < 0.05; rGLI1 = 0.395, p < 0.05, **Table S4**, **Figure 5C**). We also verified the relationship between PRKCI and the Hh pathway *via* IHC staining of xenografts (**Figure 5D**). We performed Western blotting and qRT-PCR to assess the PRKCI and Hh pathway components SMO, HHAT, and GLI1 by knocking down and upregulating PRKCI expression and the changes in the expression of SMO, HHAT, and GLI1 with the PRKCI level (**Figures 5E, F**). To further determine whether the insensitivity to radiotherapy mediated by RKCI was dependent on the Hh/GLI1 pathway, we knocked down GLI1 in the HeLa-ov-PRKCI and SiHa-ov-PRKCI cells and compared the proliferation curves. Knocking down GLI1 decreased the cell proliferation by CCK-8 assays (p < 0.05 in all groups; **Figure 6A**). However, GLI1 knockdown did not decrease the expression of PRKCI by Western blotting (p > 0.05 in all groups; **Figure 6B**). Therefore, PRKCI functions upstream of the Hh/GLI1 pathway to regulate radiosensitivity in CC cells.

Tumor growth involves enhancing Hh signaling *via* the transcription factor GLI1 (27, 28). The localization of GLI1 is related to radiotherapy and chemotherapy (29; 30). Therefore, we studied whether the intracellular localization of GLI1 was altered under the regulation of PRKCI. Through immunofluorescence assays, we analyzed and identified alterations in intracellular GLI1 localization in different groups 48 h after 8-Gy irradiation. GLI1 localized primarily to the nucleus but exhibited predominant cytosolic localization in the shRNA-PRKCI group. Conversely, GLI1 fluorescence was stronger in the nucleus in the ov-PRKCI group than in the ov-con group (**Figure 6C**). However, the intracellular localization of GLI1 was not changed in the different groups without irradiation (**Figure S4B**).

Previous reports show that PRKCI not only mediates the classic Hh/GLI1 pathway in LSCC (19) but also directly mediates the nuclear activation of GLI1 (27). Western blotting assays revealed that the expression of p-GLI1 varied according to the inhibition or overexpression of PRKCI *in vivo* (**Figure 6D**). Subsequently, we found that the changes in the protein expression level of p-GLI1 were almost undetectable in the cytoplasm, while p-GLI1 expression in the nucleus changed with the inhibition or overexpression of PRKCI in cells (**Figure 6E**). Collectively, this demonstrates that PRKCI could phosphorylate and activate GLI1 in the nucleus to affect radiosensitivity in CC cells.

### 3.6 Auranofin Enhances Radiosensitivity in Cervical Cancer Cells *In Vitro* and *In Vivo*

**Figure 7:**
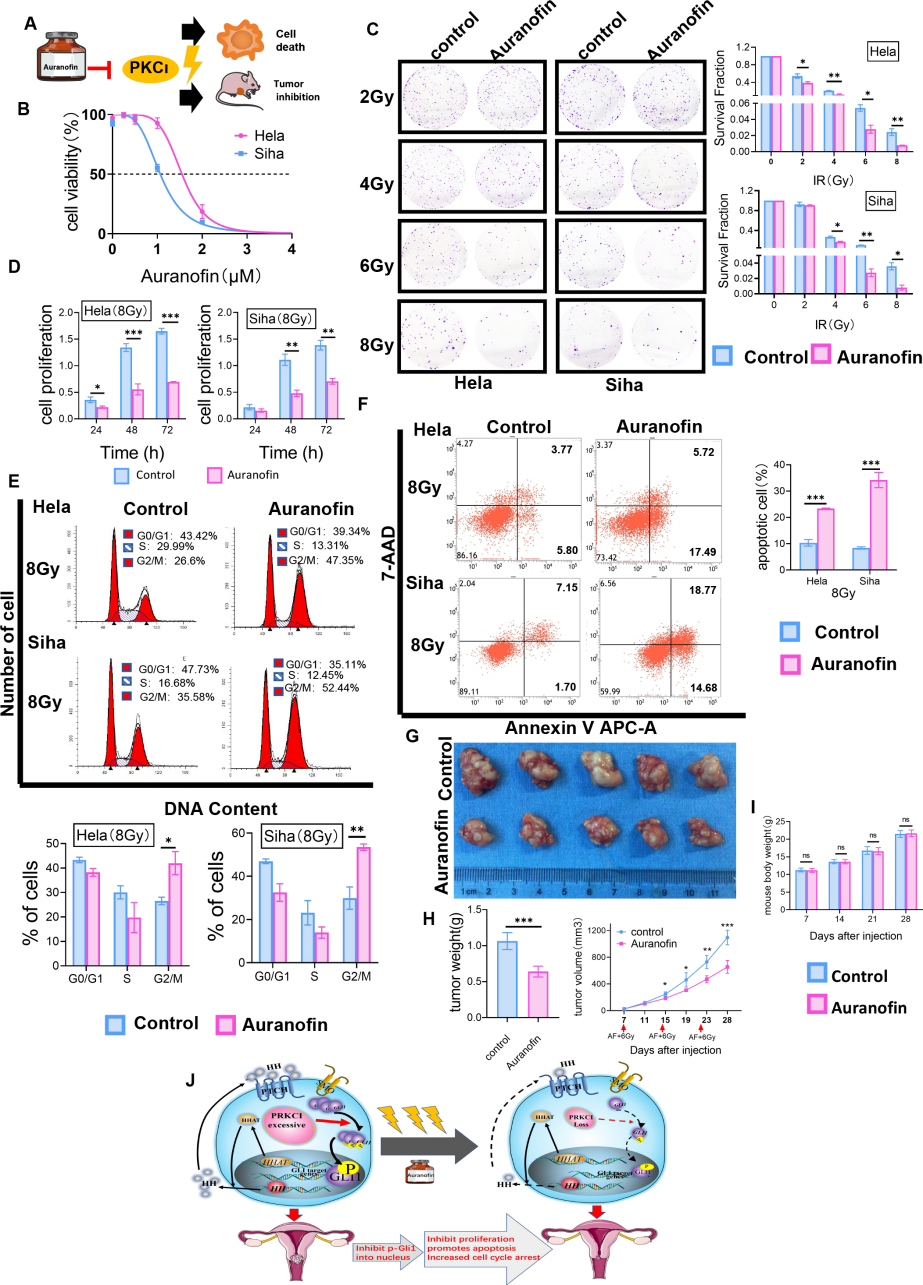
AF, a selective inhibitor of PKCι, affected radiosensitivity in CC cells *in vitro* and *in vivo*. **(A)** Workflow for AF-mediated radiosensitivity of CC *in vitro* and *in vivo*. **(B)** Representative IC50 curves calculated for AF in HeLa and SiHa cells are shown. **(C)** In colony formation assays, AF affected the proliferation of HeLa and SiHa cells treated with radiotherapy in a dose-dependent manner. **(D)** The cell viability of AF-treated HeLa and SiHa cells after radiotherapy at 8 Gy by CCK-8 assays. **(E)** AF-induced G2/M-phase cell cycle arrest with radiotherapy. **(F)** AF increased apoptosis of HeLa and SiHa cells treated with radiotherapy. **(G)** Nude mouse models were treated by intraperitoneal injection with AF or 0.1% DMSO before irradiation. **(H)** The xenograft weights and volumes were measured (n = 5/group). **(I)** Mice from two groups (AF group and control group, 5 mice per group) were weighed during the course of treatments. **(J)** A cartoon model indicating the role of PRKCI as an oncogene regulates radiosensitivity by modulating GLI1 relocalization and phosphorylation in CC *via* the Hh/GLI1 pathway. Data are shown as the mean from three independent experiments. *p < 0.05; **p < 0.01; ***p < 0.001 by multiple t-tests **(C, D, G, I)**, by unpaired t-tests **(E, F, H)** ns,not significant. AF, auranofin; CC, cervical cancer; IC50, half-maximal inhibitory concentration; CCK-8, Cell Counting Kit-8; DMSO, dimethyl sulfoxide. ns, not significant.

We investigated the effect of AF, a selective inhibitor of PKCι, on CC radiosensitivity *in vitro* and *in vivo*. A flowchart of the AF study process is shown (**Figure 7A**). First, HeLa and SiHa cells were treated with different concentrations of AF (0–4 µmol/L) for 48 h, and then cell viability was detected using the CCK‐8 assay (**Figure 7B**). The IC50 values of AF for HeLa and SiHa cells were 1.58 and 1.01 µM, respectively. AF was administered 2 h before irradiation. The colony formation assay revealed that the AF-treated groups showed significant decreases in colony formation versus the control group (containing 0.1% DMSO, negative control) with an increase in the radiotherapy dose (**Figure 7C**). Compared with the control group, the AF-treated groups showed a significant decrease in HeLa and SiHa cell viability after exposure to 8-Gy irradiation (according to the CCK-8 assay) (**Figure 7D**). Furthermore, the AF-treated groups showed increased G2/M phase arrest, in contrast to the control group, 48 h after 8-Gy irradiation (**Figure 7E**). Similarly, markedly increased apoptotic rates were observed in the AF-treated groups (**Figure 7F**). Overall, AF increased the radiosensitivity of CC cells in terms of proliferation, apoptosis, and cell cycle progression.

Our *in vitro* data on cells prompted us to study the effect of the AF/radiotherapy combination *in vivo*. Mice bearing SiHa xenografts were treated with 0.1% DMSO (control group) or 10 mg/kg of AF before radiation. Notably, the tumor volume of the control group was significantly larger than that of the AF-treated group from Day 15 (Day 15, 248.43 ± 32.20 vs. 186.16 ± 9.29 mm^3^; Day 19, 462.32 ± 106.71 vs. 308.22 ± 20.77 mm^3^; Day 23, 730.21 ± 98.12 vs. 471.42 ± 51.48 mm^3^; Day 28, 1,095.79 ± 108.03 vs. 655.88 ± 96.10 mm^3^, p < 0.001; **Figure 7G**). After 4 weeks, the weight of the isolated tumor in the control group was heavier than that in the AF-treated group (1.06 ± 0.12 vs. 0.64 ± 0.08 g, p < 0.001) (**Figure 7H**), and there was no significant difference in body weight between the two groups (p > 0.05, **Figure 7I**). All treatment regimens were tolerated and resulted in no weight loss. Overall, these data demonstrated that AF can efficiently increase the radiosensitivity of CC without obvious side effects.

## Discussion

Irradiation insensitivity plays a critical role in the failure of clinical therapy among patients who undergo radical radiotherapy. However, the underlying mechanisms are only partially defined. For this reason, novel druggable targets are urgently needed to address this issue. In this study, we demonstrated that PRKCI is overexpressed and clinically important in CC patients who undergo irradiation. Moreover, PRKCI could enhance the proliferation and decrease the apoptosis of CC cells treated with radiotherapy. We further discovered that PRKCI might modulate radiosensitivity in CC by altering the localization of GLI1 *via* the Hh/GLI1 signaling pathway. Finally, we showed that the efficacy of AF to strengthen radiosensitivity is closely comparable to that of cisplatin, which is a classic radiosensitizer in CC. Strikingly, the suppression of PRKCI evidently improved CC radiosensitivity.

Chromosome 3q26 CNG is one of the most common chromosomal variations in human cancers, including CC (31) . Some studies on non-small cell lung cancer have indicated that PKCι may play a critical role in cancer stem cell (CSC) biology and 3q26 CNA as well (19, 32, 33). PRKCI functions as an oncogene and is overexpressed in multiple human tumor types (34). The overexpression of PRKCI is associated with reduced survival and a poor prognosis (14, 15). However, few studies have examined the effects of PRKCI on tumor radiosensitivity in CC. Therefore, PRKCI may be related to radiation sensitivity in CC as a potential biomarker and therapeutic target. In our study, we found that PRKCI is overexpressed in CC tissues. Higher pathological grade, recurrence rates, and CC-related mortality were noted in the high PRKCI group. High PRKCI expression resulted in poorer responses to irradiation and shorter recurrence intervals. Previous research revealed that CC patient tissue samples had higher aPKCλ/ι expression than normal epithelium, but there was on significant relationship between aPKC_λ_/ι expression and tumor progression (17). The outcomes of these studies are distinct from those of our study, likely because of the different inclusion criteria (their studies included populations that only received surgery). The patients included in our research underwent radical irradiation as their major treatment. In addition, we identified PRKCI as an independent prognostic factor in CC patients who received radiotherapy. This is the first time PRKCI was shown to be clinically related to CC in patients who underwent radiotherapy.

After analysis of clinical samples, we used experimental models to further validate our results. When DNA is damaged by irradiation, cells often activate cell cycle checkpoints at the G1-S and G2-M transitions, and regulation of many genes related to DNA damage repair is involved in radioresistance (35, 36). The impact of radiotherapy on the cell cycle is more significant (37). Irradiation eventually leads to cell G1 or G2-M phase arrest and growth suppression (2, 38). In our current research, neither the group with upregulated expression nor the group with downregulated expression exhibited differences in cell proliferation or apoptosis in the absence of radiotherapy. Interestingly, on the basis of radiotherapy, cell proliferation and sensitivity to radiotherapy remained consistent with the PRKCI level, and the previous findings were validated in the xenograft models. For the above observation concerning whether radiotherapy affects the impact of PRKCI changes, the two following explanations can be considered. One is that irradiation promotes the activation or acquisition of CSCs by regulating multiple signaling mechanisms (39). Another is that dying tumor cells after radiation stimulate the growth of living tumor cells (40). Some studies have indicated that PKCι may play a key role in CSC biology (33, 41). Additionally, oncogenic PKCι mediates multiple signaling mechanisms that promote the survival of different tumor cell types (34). For the first time, we identified a novel role of PRKCI in mediating CC radiosensitivity, that PRKCI may be a crucial regulator in the DNA damage repair process. These results demonstrated that PRKCI mediates cell cycle arrest and apoptosis to change the proliferation latency associated with radiosensitivity in CC.

An abnormal Hh signaling pathway has been detected in various human malignant tumors (42). The Hh signaling pathway plays an essential role in disease pathogenesis and mediates the development of radioresistance in CC (43, 44). Previously, PRKCI was shown to activate Hh signaling in LSCC and BCC (19, 27). Hedgehog acyltransferase (HHAT) functions as a palmitoyl acyltransferase for the Hh and Sonic Hedgehog (Shh) family of proteins (45, 46). SMO, a signal transduction protein, transduces signals to other proteins after activation. GLI1, an oncogenic transcription factor, is activated and free from protein complexes, and activated GLI1 is ultimately an effector of the Hh pathway. Eventually, GLI1 enters the nucleus and directly regulates the expression of various target genes (28, 47). In our study, IHC of PRKCI, SMO, and GLI1 showed a tight interrelation between the Hh/GLI1 pathway and PRKCI. Subsequently, we found that the expression of HHAT, SMO, and GLI1 changed consistently with the downregulation and upregulation of PRKCI levels after radiotherapy, which was validated *in vitro* and *in vivo*.

Finally, we detected a novel mechanism by which PRKCI regulates the subcellular relocalization of GLI1 in CC cells. At present, there are few studies about the relocalization of GLI1 after radiotherapy treatment. Yao et al. (30) reported that GLI1 was abundant in the nucleus and cytoplasm of Eca109R cells in esophageal cancer, overexpression of GLI1 in Eca109 cells led to decreased levels of radiosensitivity, and immunofluorescence revealed GLI1 protein aggregation around the nucleus. The outcomes of this study were the same as ours in terms of PRKCI altering the localization of GLI1 after radiotherapy. In addition, aspirin sensitizes malignant glioma cells to temozolomide therapy by inhibiting the SHH/GLI1 signaling pathway, preventing most GLI1 translocation into the nucleus and resulting in higher expression levels in the cytoplasm (29). Our findings are consistent with these previous observations that the changes in GLI1 localization affect the sensitivity to radiotherapy. Scott et al. reported that aPKC mediates Hh signaling by phosphorylating and activating GLI1 in the growth of BCC (27). Doheny et al. summarized the known target genes of GLI1 in human cancers, including components of the Hh signaling pathway (PTCH, SHH, and SMO) (28). However, one limitation of our study is that we did not verify whether the Hh components are target genes of GLI1. Here, these findings demonstrated that PRKCI attenuates radiosensitivity by mediating the Hh signaling pathway in CC cells, increasing nuclear GLI1 localization and phosphorylation.

Collectively, the above findings suggest that PRKCI might be a critical regulatory factor for radiosensitivity and that targeting PRKCI might represent a new therapeutic strategy for improving radiosensitivity in CC patients. AF is a specific thioredoxin reductase (TRXR) inhibitor that has been recently repurposed as a potential anticancer medication (48, 49). Moreover, AF has shown a favorable safety profile (50). A previous study suggested that AF is a selective inhibitor of the PKCι oncogene (41). Oral dosing of AF was also included in a clinical trial to evaluate its benefit in asymptomatic ovarian cancer patients (51). Herein, we uncovered that AF could increase the radiosensitivity of CC cells, showing anticancer efficacy in terms of proliferation and apoptosis. Additionally, AF could induce cell cycle arrest at the G2/M phase. Raninga et al. (52) previously found that AF could inhibit triple-negative breast cancer *in vivo* and *in vitro*, consistent with our results. Interestingly, AF showed efficacy as a chemotherapeutic drug in a previous study, while we studied its radiosensitizing properties in this study. Overall, our results indicate that AF can act as an effective radiosensitizer in CC. Nag et al. (53) reported that AF protects the intestinal tract from radiation damage by modulating the p53/21 signaling pathway and sensitizing colon tumors to radiation. Taken together with other previous studies, this shines a new light on this well-established anticancer drug and supports the use of AF as a radiosensitizer in CC patients. In the future, we believe that drug repositioning research will boost anticancer therapy by uncovering new applications for existing drugs.

## Conclusion

In summary, we showed that a novel oncogene, PRKCI, acts as an independent prognostic factor for CC patients who have undergone irradiation and attenuates the radiosensitivity of CC both *in vitro* and *in vivo*. We also found that PRKCI is a regulator reliant on the Hh/GLI1 signaling pathway that mediates radiotherapy-triggered cell apoptosis, proliferation, and cell cycle arrest (**Figure 7J**). The results linking PRKCI to CC radiosensitivity indicate that PRKCI inhibition may be a promising approach for CC treatment in the clinic, particularly for patients who are insensitive to radiotherapy.

## Data Availability Statement

Transcriptome RNA-seq data and the CNA status of PRKCI in TCGA CESC samples were analyzed with cBioPortal software (http://www.cbioportal.org/). The raw data supporting the conclusions of this article will be made available by the authors, without undue reservation.

## Ethics Statement

The studies involving human participants were reviewed and approved by the Research Ethics Committee of The Second Affiliated Hospital of Fujian Medical University. The patients/participants provided their written informed consent to participate in this study. The animal study was reviewed and approved by the Animal Experiment Ethics Committee (Sun Yat-Sen University, China). Written informed consent was obtained from the individual(s) for the publication of any potentially identifiable images or data included in this article.

## Author Contributions

ZW, CH, HWL, and ZL planned the experiments and prepared the manuscript. ZW conducted most of the experiments, collected the data, analyzed the results, and wrote most of the manuscript. CH performed the irradiation-related experiments. RL and HL performed some of the animal studies. All authors read and approved the final paper.

## Funding

This work was financially supported by grants from the National Natural Science Foundation of China (NSFC) (No. 81972433) to ZL and the Fujian Provincial Health Technology Project (No. 2019-1-15).

## Conflict of Interest

The authors declare that the research was conducted in the absence of any commercial or financial relationships that could be construed as a potential conflict of interest.

## Publisher’s Note

All claims expressed in this article are solely those of the authors and do not necessarily represent those of their affiliated organizations, or those of the publisher, the editors and the reviewers. Any product that may be evaluated in this article, or claim that may be made by its manufacturer, is not guaranteed or endorsed by the publisher.

## Supplementary Material

Supplementary Figure 1
**(A)** ShRNA-PRKCI (2) and ShRNA-PRKCI (1) were selected and screened for downregulation in HeLa and SiHa cells by qRT-PCR. **(B)** Ov-PRKCI was selected and screened for upregulation in HeLa and SiHa cells by qRT-PCR. **(C)** ShRNA-PRKCI and ov-PRKCI were verified for the downregulation and upregulation in HeLa and SiHa cells by Western blotting analysis. **(D, E)** qRT-PCR and Western blotting assays were applied to assess the reduction in GLI1 mRNA and protein expression after 48 and 72 h of transfection in the HeLa-ov-PRKCI and SiHa-ov-PRKCI cells. Data are shown as the mean ± SD from three independent experiments. *P < 0.05; **P < 0.01; ***P < 0.001; ****P < 0.0001 by unpaired t test **(A**–**E)**, NS, not significant.

Supplementary Figure 2
**(A)** Cell viability of the shRNA-PRKCI- and ov-PRKCI-transfected HeLa and SiHa cells with no radiotherapy determined by CCK-8 assays. **(B)** The influence of PRKCI suppression and overexpression have no effect on apoptosis of HeLa and SiHa cells without radiotherapy. Data are shown as the mean ± SD from three independent experiments. NS, not significant.

Supplementary Figure 3
**(A)** Influence of PRKCI suppression on the cell cycle progression of HeLa and SiHa cells without radiotherapy. **(B)** Influence of PRKCI overexpression on the cell cycle progression of HeLa and SiHa cells without radiotherapy. Data are shown as the mean ± SD from three independent experiments. NS, not significant.

Supplementary Figure 4
**(A)** Original pictures of xenografts from different groups. **(B)** The relative protein localization of GLI1 after transfection with shRNA-con and shRNA-PRKCI or ov-con and ov-PRKCI in HeLa and SiHa cells without radiation was determined by immunofluorescence assays. Scale bars are shown.

Supplementary Figure 5
**(A)** AF affect the cell viability of HeLa and SiHa cells treated with radiotherapy at 0Gy as shown by CCK-8 assays. **(B)** The cell viability of AF-treated HeLa and SiHa cells with and without irradiation by CCK-8 assays. Data are shown as the mean ± SD. Data are shown as the mean from three independent experiments. *P < 0.05; **P < 0.01; ***P < 0.001 by unpaired t tests.

Supplementary Figure 6
**(A)** AF-induced G2/M-phase cell cycle arrest treated without radiation. **(B)** G2/M phase arrest of AF-treated HeLa and SiHa cells with and without irradiation. Data are shown as the mean ± SD. Data are shown as the mean from three independent experiments. *P < 0.05; **P < 0.01 by unpaired t tests.

Supplementary Figure 7
**(A)** AF increased apoptosis of HeLa and SiHa cells without radiotherapy. **(B)** The cell apoptosis of AF-treated HeLa and SiHa cells with and without irradiation. Data are shown as the mean ± SD. Data are shown as the mean from three inde-pendent experiments. *P < 0.05; **P < 0.01 by unpaired t tests.

Supplementary Table 1 Oligonucleotide sequences for this study.

Supplementary Table 2 SiRNAs targeting human GLI1 sequences for this study.

Supplementary Table 3 Primers used in PCR.

Supplementary Table 4 Correlation of PRKCI and SMO/GLI1 expression in CC.
