# Correction: PRKCI mediates radiosensitivity *via* the Hedgehog/GLI1 pathway in cervical cancer

**DOI:** 10.3389/fonc.2026.1765010

**Published:** 2026-01-13

**Authors:** Zhuna Wu, Chunxian Huang, Ruixin Li, Hui Li, Huaiwu Lu, Zhongqiu Lin

**Affiliations:** 1 Department of Gynecological Oncology, Sun Yat-Sen Memorial Hospital, Sun Yat-Sen University, Guangzhou, China; 2 Guangdong Provincial Key Laboratory of Malignant Tumor Epigenetics and Gene Regulation, Sun Yat-sen Memorial Hospital, Sun Yat-sen University, Guangzhou, China; 3 Department of Gynecology and Obstetrics, The Second Affiliated Hospital of Fujian Medical University, Fujian Medical University, Quanzhou, China

**Keywords:** PRKCI, radiosensitivity, Hedgehog/GLI1 pathway, cervical cancer, auranofin

There was a mistake in **Figure 7C** as published. The panels representing SiHa cells treated with Auranofin at 6 Gy and 8 Gy radiation doses were inadvertently duplicated during the figure assembly process. This duplication was unintentional and does not reflect the underlying experimental data, which remain valid and consistent with the original findings. The corrected **Figure 7C** appears below. The panels for 6 Gy and 8 Gy Auranofin treatment in SiHa cells are distinct and accurately represent the original exp erimental results. No other text, data, figures, or conclusions in the manuscript are affected by this change.

**Figure 7:**
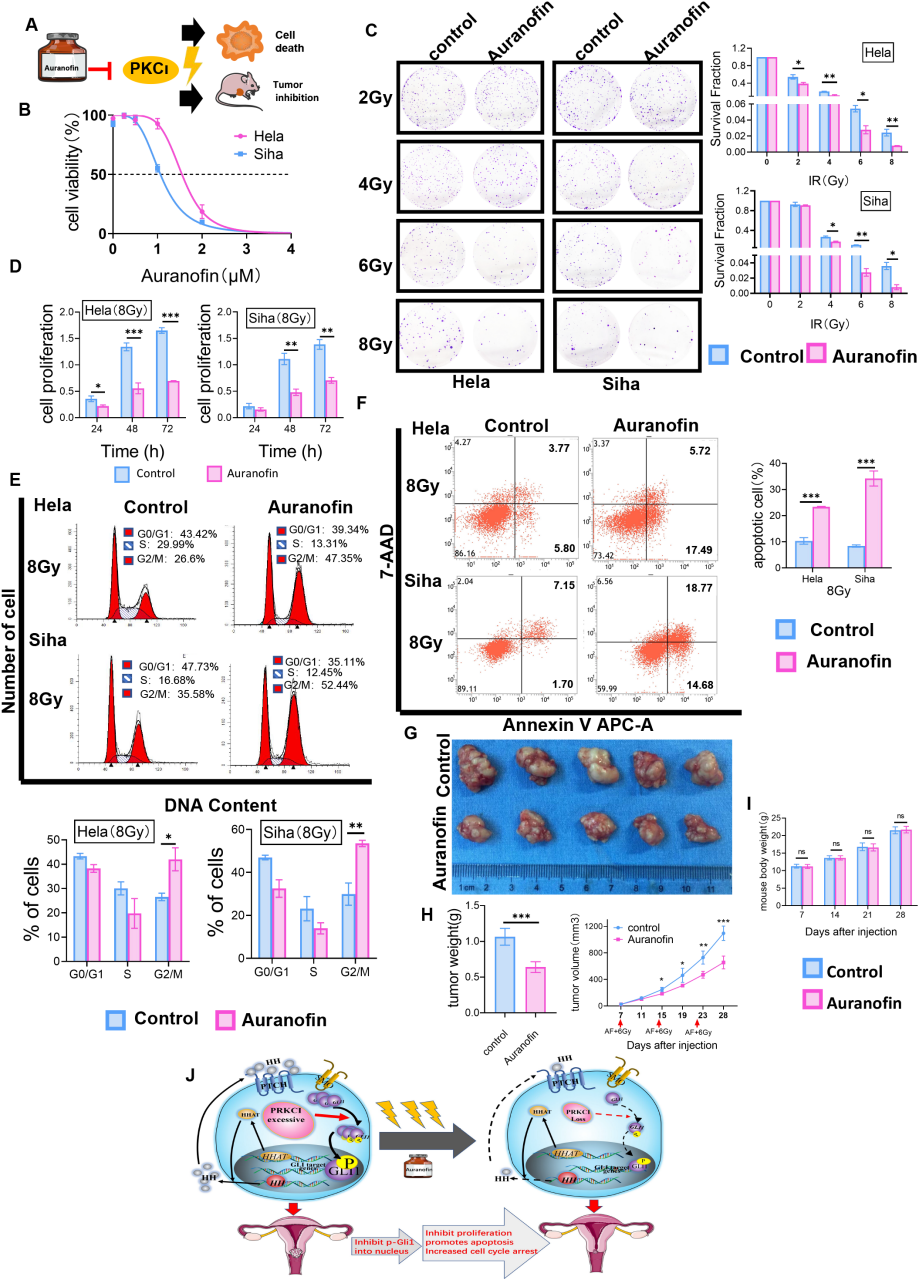
AF, a selective inhibitor of PKC_I_, affected radiosensitivity in CC cells *in vitro* and *in vivo*. **(A)** Workflow for AF-mediated radiosensitivity of CC *in vitro* and *in vivo*. **(B)** Representative IC50 curves calculated for AF in HeLa and SiHa cells are shown. **(C)** In colony formation assays, AF affected the proliferation of HeLa and SiHa cells treated with radiotherapy in a dose-dependent manner. **(D)** The cell viability of AF-treated HeLa and SiHa cells after radiotherapy at 8 Gy by CCK-8 assays. **(E)** AF-induced G2/M-phase cell cycle arrest with radiotherapy. **(F)** AF increased apoptosis of HeLa and SiHa cells treated with radiotherapy. **(G)** Nude mouse models were treated by intraperitoneal injection with AF or 0.1% DMSO before irradiation. **(H)** The xenograft weights and volumes were measured (n = 5/group). **(I)** Mice from two groups (AF group and control group, 5 mice per group) were weighed during the course of treatments. **(J)** A cartoon model indicating the role of PRKCI as an oncogene regulates radiosensitivity by modulating GLI1 relocalization and phosphorylation in CC via the Hh/GLI1 pathway. Data are shown as the mean from three independent experiments. *p < 0.05; **p < 0.01; ***p < 0.001 by multiple t-tests **(C, D, G, I)**, by unpaired t-tests **(E, F, H)** ns,not significant. AF, auranofin; CC, cervical cancer; IC50, half-maximal inhibitory concentration; CCK-8, Cell Counting Kit-8; DMSO, dimethyl sulfoxide. ns, not significant.

The original version of this article has been updated.

