# Supplementary figures and images for: PRKCI Mediates Radiosensitivity via the Hedgehog/GLI1 Pathway in Cervical Cancer
Source: Front Oncol. 2022 Jun 16;12:887139. doi: 10.3389/fonc.2022.887139 (PMC9243290; doi:10.3389/fonc.2022.887139)

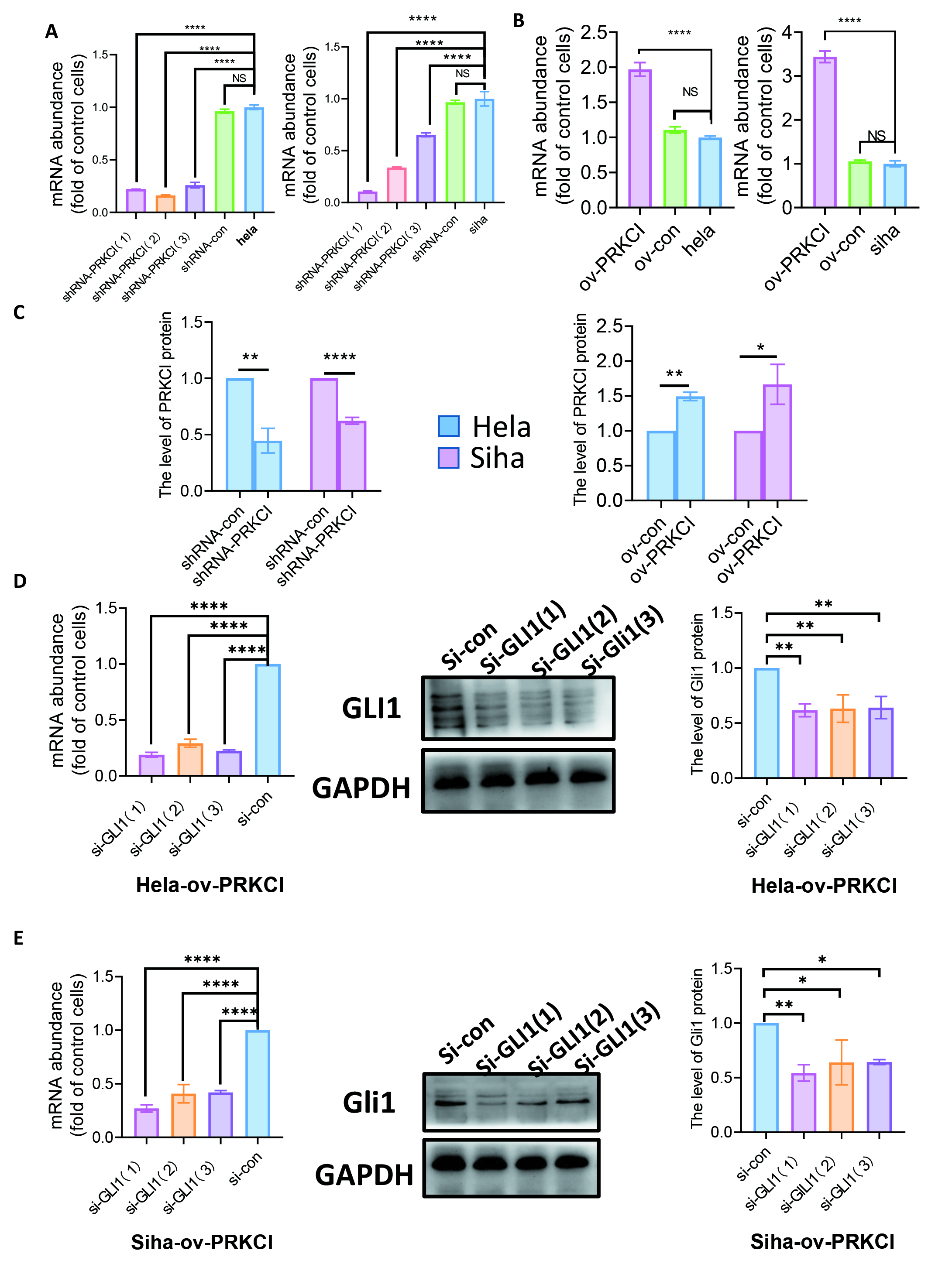

Supplement: Supplementary Figure 1 — (A) ShRNA-PRKCI (2) and ShRNA-PRKCI (1) were selected and screened for downregulation in HeLa and SiHa cells by qRT-PCR. (B) Ov-PRKCI was selected and screened for upregulation in HeLa and SiHa cells by qRT-PCR. (C) ShRNA-PRKCI and ov-PRKCI were verified for the downregulation and upregulation in HeLa and SiHa cells by Western blotting analysis. (D, E) qRT-PCR and Western blotting assays were applied to assess the reduction in GLI1 mRNA and protein expression after 48 and 72 h of transfection in the HeLa-ov-PRKCI and SiHa-ov-PRKCI cells. Data are shown as the mean ± SD from three independent experiments. *P < 0.05; **P < 0.01; ***P < 0.001; ****P < 0.0001 by unpaired t test (A–E), NS, not significant. [file Image_1.tif]

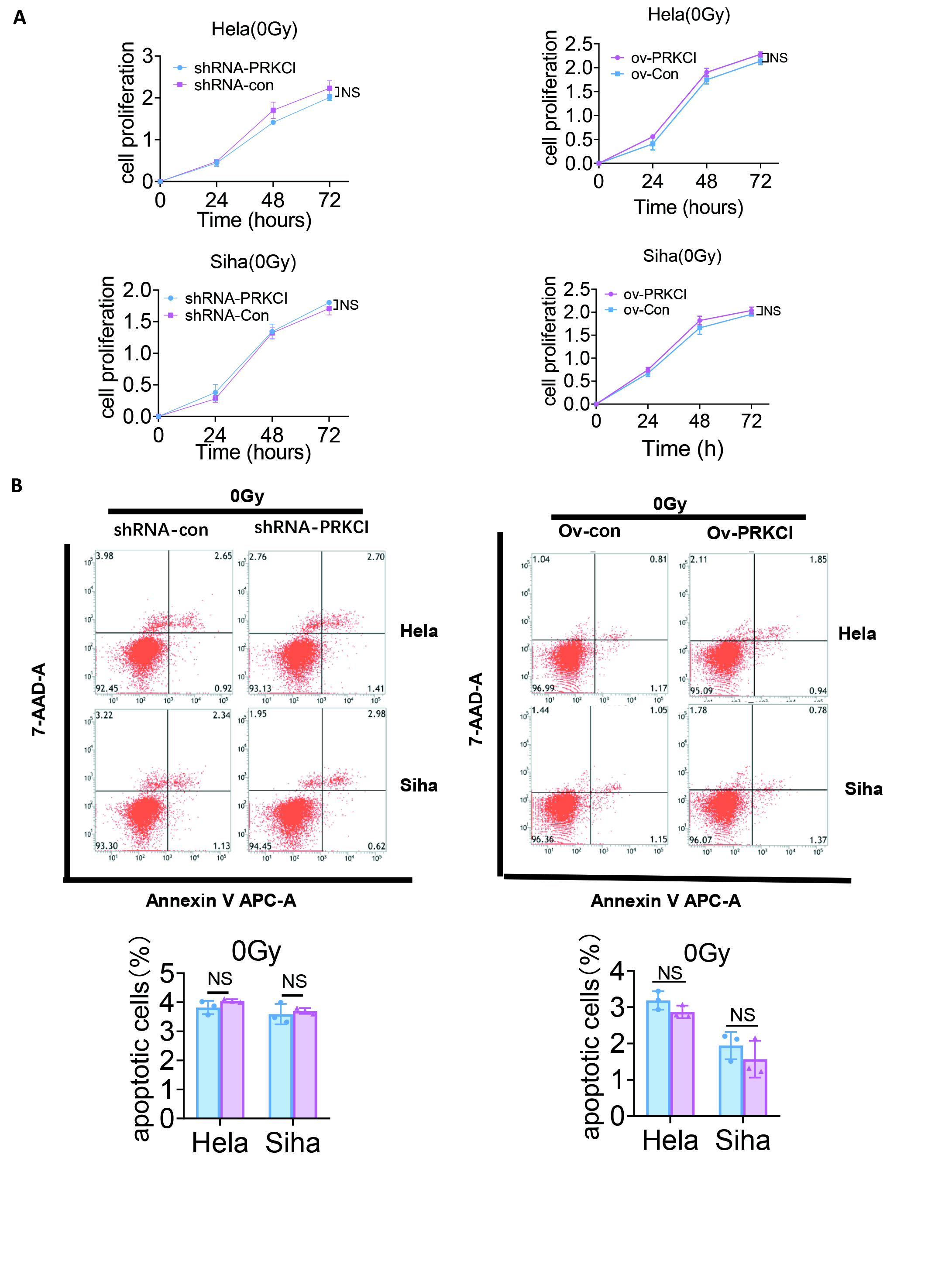

Supplement: Supplementary Figure 2 — (A) Cell viability of the shRNA-PRKCI- and ov-PRKCI-transfected HeLa and SiHa cells with no radiotherapy determined by CCK-8 assays. (B) The influence of PRKCI suppression and overexpression have no effect on apoptosis of HeLa and SiHa cells without radiotherapy. Data are shown as the mean ± SD from three independent experiments. NS, not significant. [file Image_2.tif]

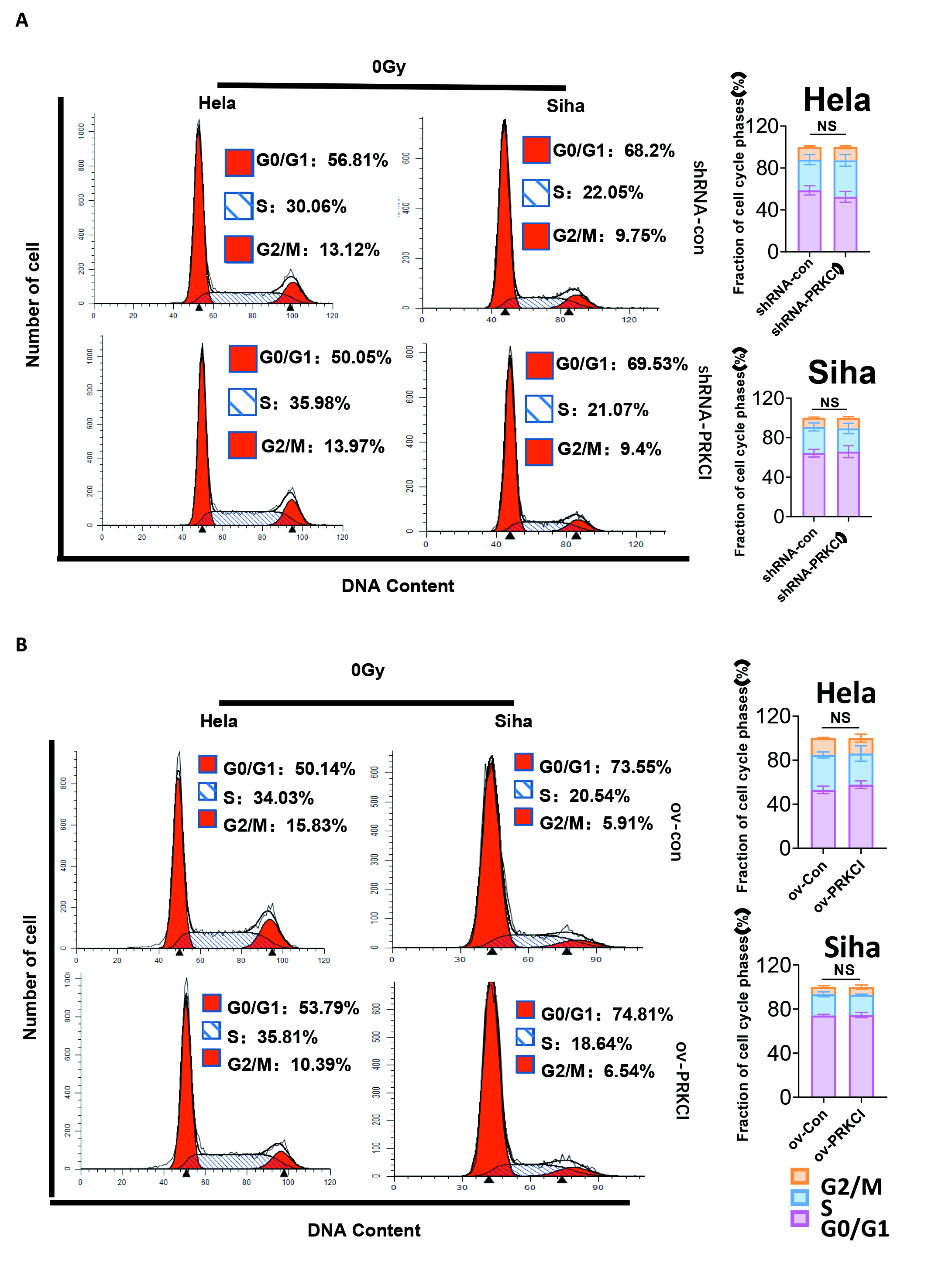

Supplement: Supplementary Figure 3 — (A) Influence of PRKCI suppression on the cell cycle progression of HeLa and SiHa cells without radiotherapy. (B) Influence of PRKCI overexpression on the cell cycle progression of HeLa and SiHa cells without radiotherapy. Data are shown as the mean ± SD from three independent experiments. NS, not significant. [file Image_3.tif]

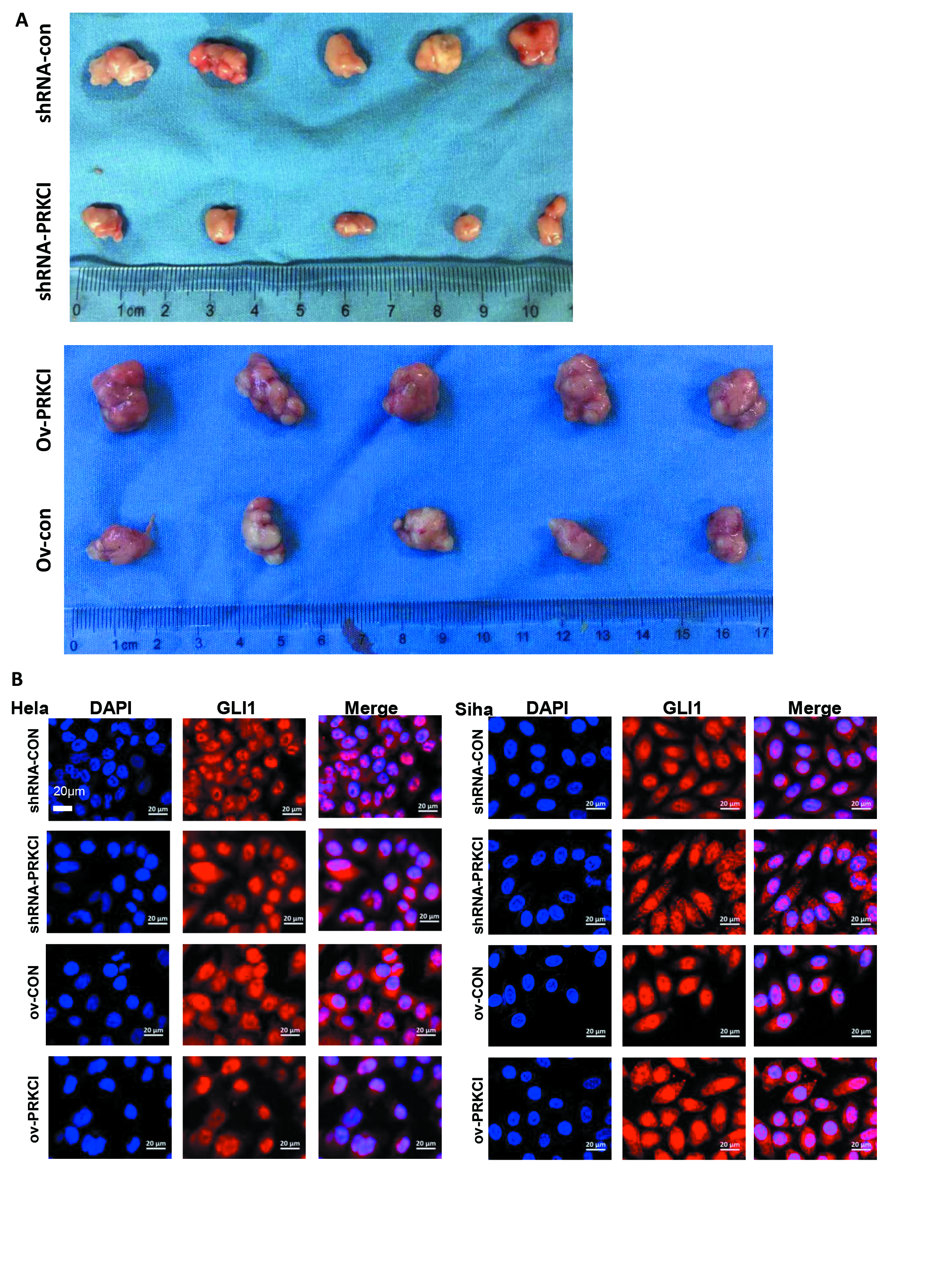

Supplement: Supplementary Figure 4 — (A) Original pictures of xenografts from different groups. (B) The relative protein localization of GLI1 after transfection with shRNA-con and shRNA-PRKCI or ov-con and ov-PRKCI in HeLa and SiHa cells without radiation was determined by immunofluorescence assays. Scale bars are shown. [file Image_4.tif]

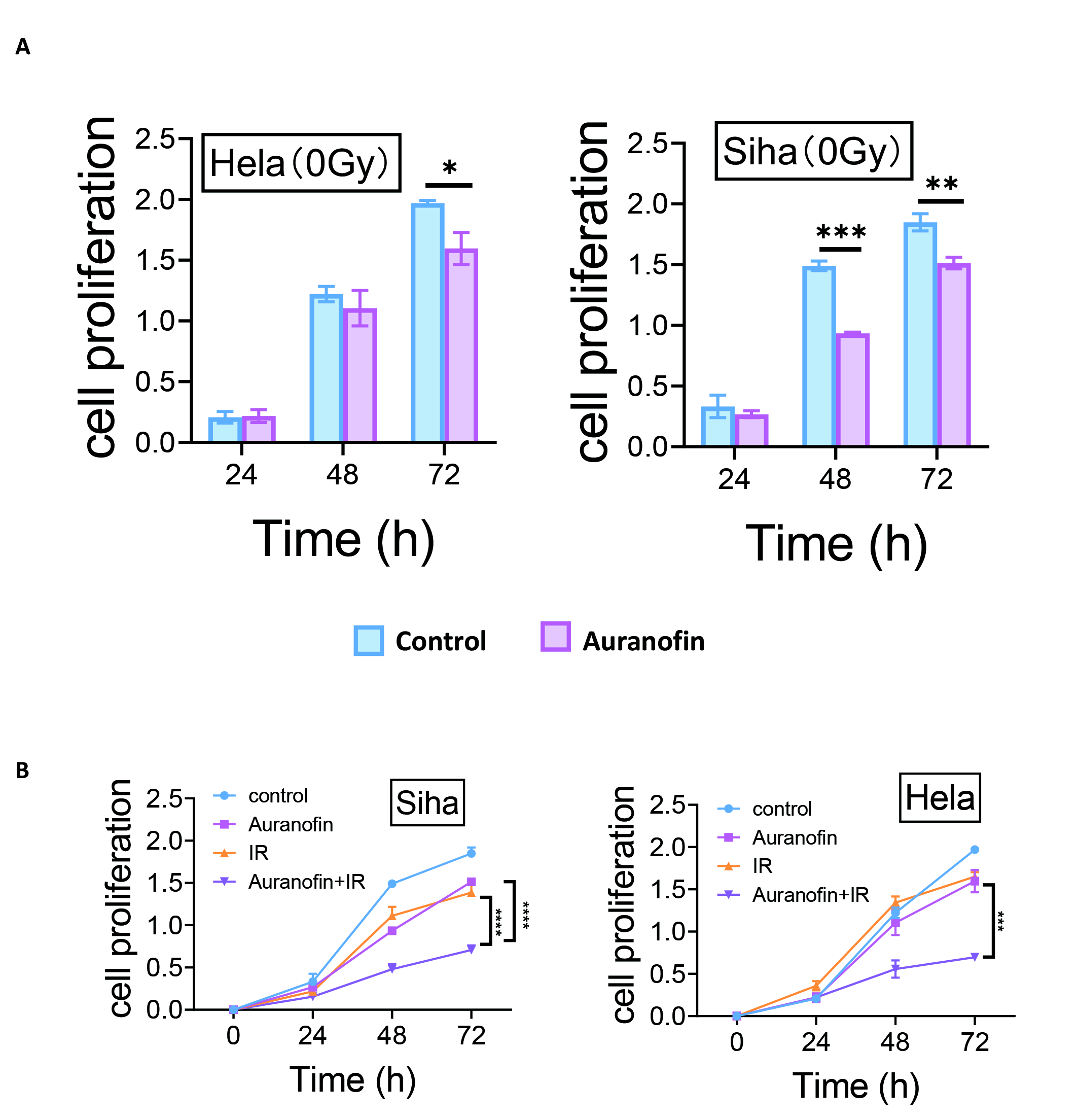

Supplement: Supplementary Figure 5 — (A) AF affect the cell viability of HeLa and SiHa cells treated with radiotherapy at 0Gy as shown by CCK-8 assays. (B) The cell viability of AF-treated HeLa and SiHa cells with and without irradiation by CCK-8 assays. Data are shown as the mean ± SD. Data are shown as the mean from three independent experiments. *P < 0.05; **P < 0.01; ***P < 0.001 by unpaired t tests. [file Image_5.tif]

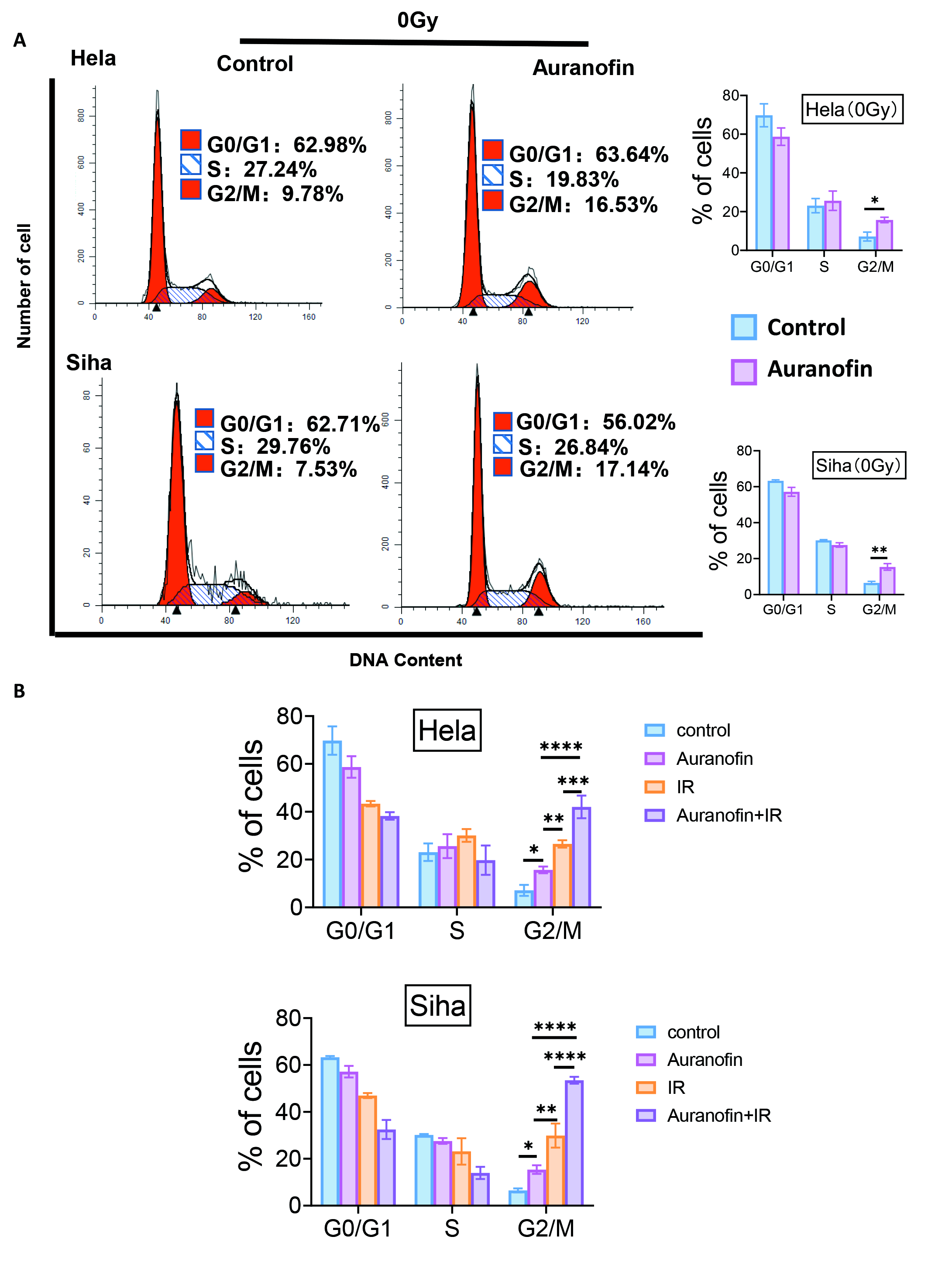

Supplement: Supplementary Figure 6 — (A) AF-induced G2/M-phase cell cycle arrest treated without radiation. (B) G2/M phase arrest of AF-treated HeLa and SiHa cells with and without irradiation. Data are shown as the mean ± SD. Data are shown as the mean from three independent experiments. *P < 0.05; **P < 0.01 by unpaired t tests. [file Image_6.tif]

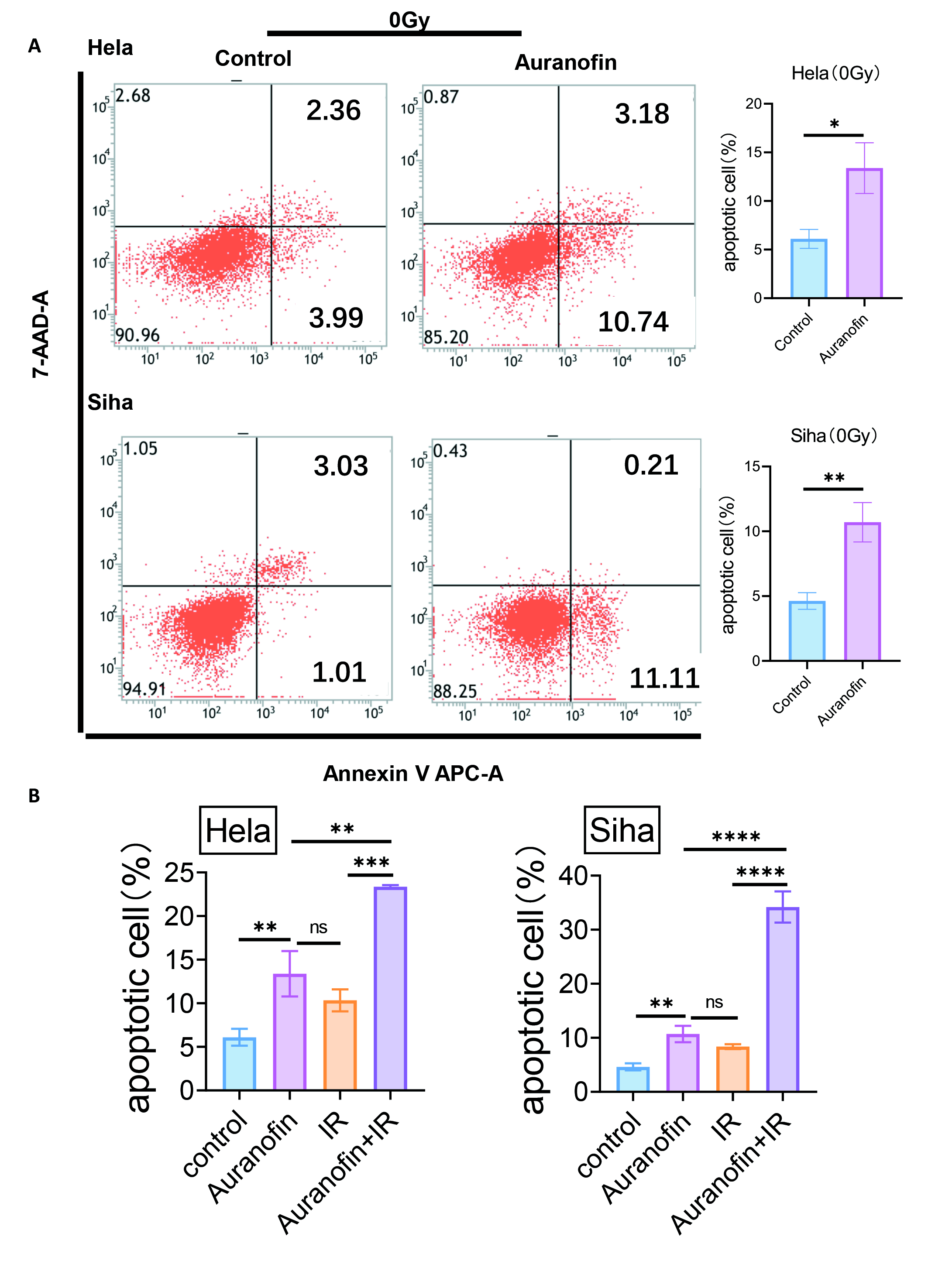

Supplement: Supplementary Figure 7 — (A) AF increased apoptosis of HeLa and SiHa cells without radiotherapy. (B) The cell apoptosis of AF-treated HeLa and SiHa cells with and without irradiation. Data are shown as the mean ± SD. Data are shown as the mean from three inde-pendent experiments. *P < 0.05; **P < 0.01 by unpaired t tests. [file Image_7.tif]
