# Supplementary material for: PRKCI Mediates Radiosensitivity via the Hedgehog/GLI1 Pathway in Cervical Cancer
Source: Front Oncol. 2022 Jun 16;12:887139. doi: 10.3389/fonc.2022.887139 (PMC9243290; doi:10.3389/fonc.2022.887139)
Supplement: Supplementary Table 1 — Oligonucleotide sequences for this study. [file Table_1.doc]

**Table S1. Oligonucleotide sequences for this study.**

| **Name** | **Sequence** |
| --- | --- |
| **sh-RNA** |  |
| **sh-PRKCI(1)**  **most efficiency in SiHa** | 5′-TGAAGAACATGCCAGATTT-3′ |
| **sh-PRKCI(2)**  **most efficiency in HeLa** | 5′-AGTCTAGGTCTTCAGGATT-3′ |
| sh-PRKCI(3) | 5’-TTTAGACTTTATGAGCTAA-3’ |
| sh-NC | 5′-TTCTCCGAACGTGTCACGT-3′ |
