# Supplementary material for: PRKCI Mediates Radiosensitivity via the Hedgehog/GLI1 Pathway in Cervical Cancer
Source: Front Oncol. 2022 Jun 16;12:887139. doi: 10.3389/fonc.2022.887139 (PMC9243290; doi:10.3389/fonc.2022.887139)
Supplement: Supplementary Table 2 — SiRNAs targeting human GLI1 sequences for this study. [file Table_2.doc]

**Table S2. siRNAs targeting human Gli1 sequences for this study.**

| **Name** | **Sequence** |
| --- | --- |
| **si-RNA** |  |
| **si-Gli1(1)**  **most efficiency** | Sense：CCAGGAAUUUGACUCCCAATT  Antisense: UUGGGAGUCAAAUUUCCUGGTT |
| si-Gli1(2) | Sense：CCGAGUAUCCAGGAUACAATT  Antisense: UUGUAUCCUGGAUACUCGGTT |
| si-Gli1(3) | Sense：GCUGGACUUUGUGGCUAUUTT  Antisense: AAUAGCCACAAAGUCCAGCTT |
| si-con | Sense：UUCUCCGAACGUGUCACGUTT  Antisense: ACGUGACACGUUCGGAGAATT |
