# Supplementary material for: PRKCI Mediates Radiosensitivity via the Hedgehog/GLI1 Pathway in Cervical Cancer
Source: Front Oncol. 2022 Jun 16;12:887139. doi: 10.3389/fonc.2022.887139 (PMC9243290; doi:10.3389/fonc.2022.887139)
Supplement: Supplementary Table 3 — Primers used in PCR. [file Table_3.doc]

Table S3: Primers used in PCR

| Gene | sense | antisense |
| --- | --- | --- |
| GAPDH | 5′-GCACCGTCAAGGCTGAGAAC-3′ | 5′- TGGTGAAGACGCCAGTGGA-3′ |
| PRKCI | 5′-GCCAGGAGATACAACCAGCAC-3′ | 5′-CAAGAGCCCACCAGTCAACAC-3′ |
| HHAT | 5′-CTCAGCAGCTGTCTTGGTAAAGG-3′ | 5′-TGAAGGTTTACTGCGTTCATTTAGG-3′ |
| SMO | 5′-CCTTCAGCTGCCACTTCTACGAC-3′ | 5′- TCGGGCGATTCTTGATCTCAC -3′ |
| Gli1 | 5’-GAGCACGAGGGCTGCAGTAA-3′ | 5′- TCGCAGCGAGCTAGGATCTGTA -3′ |
