# Supplementary material for: PRKCI Mediates Radiosensitivity via the Hedgehog/GLI1 Pathway in Cervical Cancer
Source: Front Oncol. 2022 Jun 16;12:887139. doi: 10.3389/fonc.2022.887139 (PMC9243290; doi:10.3389/fonc.2022.887139)
Supplement: Supplementary Table 4 — Correlation of PRKCI and SMO/GLI1 expression in CC. [file Table_4.doc]

Table S4: Correlation of PRKCI and SMO/GLI1 expression in cervical cancer

|  | PRKCI | | r value^†^ | P value^†^ |
| --- | --- | --- | --- | --- |
|  | Low expression | High expression |  |  |
| SMO |  |  |  |  |
| Low expression | 7 | 7 | 0.434 | 0.039^*^ |
| High expression | 10 | 36 |  |  |
| Gli1 |  |  |  |  |
| Low expression | 7 | 6 | 0.395 | 0.021^*^ |
| High expresssion | 10 | 37 |  |  |

^†^ Chi-square test, *p < 0.05.
