# Supplementary material for: PRKCI Mediates Radiosensitivity via the Hedgehog/GLI1 Pathway in Cervical Cancer
Source: Front Oncol. 2022 Jun 16;12:887139. doi: 10.3389/fonc.2022.887139 (PMC9243290; doi:10.3389/fonc.2022.887139)
Supplement: Supplementary file 12 [file DataSheet_1.docx]

**Supplementary Methods**

**RNA extraction and quantitative real-time PCR (qRT-PCR)**

Total RNA was extracted from cultured cell lines using RNA-Quick purification Kit (Yishan Biotechnology Co, Shanghai, China) following the manufacturer's instructions. RNA concentration was measured with NanoDrop ND-2000 spectrophotometers (Life Technologies, CA, USA) and then the RNA was then transcribed to cDNA using 5 X PrimeScript RT Master Mix (Takara, Japan). Real-time PCR was performed using TB Green^®^ Premix Ex Taq™ II (Tli RNaseH Plus) (Takara, Japan) and analyzed on a CFX connectTM (Bio-Rad, USA). The results were normalized to the expression of glyceraldehyde-3-phosphate dehydrogenase (GAPDH). All the primer sequences are listed in Table S2. The qRT-PCR data were analyzed and expressed relative to CT (cycle threshold) values. The qRT-PCR results were analyzed and showed as the fold change (2^-∆∆CT^). For the analysis of the expression in cells, the levels were compared with the controls and converted to the fold change (2^-∆∆CT^). The quantitative PCR reaction for each sample was repeated in triplicate.

**Colony formation assay of Auranofin**

Cells were counted and plated in 6-well plates at different densities of 2x10^3^, 4×10^3^, 8×10^3^, 1.2×10^4^ or 1.5×10^4^ per well in Auranofin group while Cells were counted and plated in 6-well plates at different densities of 1x10^3^, 2×10^3^, 4×10^3^, 8×10^3^ or 1.2×10^4^ per well in control（0.1% DMSO）group，when irradiated with various dose (0, 2, 4, 6 and 8gy) after 24h. Cells were treated with auranofin were given 2h before starting irradiation dose. Cells were maintained in media containing 5% FBS at 37℃ and were replaced every 4 days. Fourteen days after seeding, colonies were fixed with methanol and stained with 0.5% crystal violet. Visible colonies were then utilized by image J soft to counted.

**Xenograft experiment of Auranofin**

Female BALB/c nude mice (4-5 weeks old) were used for the CC xenograft model, which were obtained from the Laboratory Animal Center (Sun Yat-Sen University, China). First, a suspension of 5×10^6^ cells was inoculated subcutaneously into right axillary region (n= 5 per group). Subsequently, one week after inoculation, Mice with tumors of 30–50 mm3 were randomly assigned to two groups, each group containing five mice. Mice were treated once a week by intraperitoneal injection for 3 weeks with vehicle control (0.1% DMSO), AF 10 mg/kg. The mice were subjected a locoregional applied body dose of 6 Gy every week using an X-RAD irradiator (Rad Source 2000, US) after injection. Tumor size was measured every 4 days, mice weight were weighed every week and tumor volume was calculated with the formula: volume = width^2^ × length × 0.5. After the 28-day experiment, all mice were sacrificed, and the tumors were excised, harvested and weighed.
